# Climate threat on the Macaronesian endemic bryophyte flora

**DOI:** 10.1038/srep29156

**Published:** 2016-07-05

**Authors:** Jairo Patiño, Rubén G. Mateo, Florian Zanatta, Adrien Marquet, Silvia C. Aranda, Paulo A. V. Borges, Gerard Dirkse, Rosalina Gabriel, Juana M. Gonzalez-Mancebo, Antoine Guisan, Jesús Muñoz, Manuela Sim-Sim, Alain Vanderpoorten

**Affiliations:** 1 Island Ecology and Evolution Research Group, Instituto de Productos Naturales y Agrobiología (IPNA-CSIC), Tenerife, Canary Islands, Spain; 2 Department of Ecology and Evolution, University of Lausanne, Lausanne, Switzerland; 3 Centre for Ecology, Evolution and Environmental Changes (cE3c)/Azorean Biodiversity Group & University of the Azores, Angra do Heroísmo, Azores, Portugal; 4 Department of Plant Biology, University of La Laguna, Tenerife, Spain; 5 Museo Nacional de Ciencias Naturales (CSIC), 28006 Madrid, Spain; 6 Natuurmuseum Nijmegen, Nijmegen, The Netherlands; 7 Institute of Earth Surface Dynamics, University of Lausanne, Lausanne, Switzerland; 8 Real Jardín Botánico (CSIC), Madrid, Spain; 9 Centre for Ecology, Evolution and Environmental Changes (cE3c), Universidade de Lisboa, Faculdade de Ciências de Lisboa, Lisboa and Museu Nacional de História Natural e da Ciência, Jardim Botânico, Lisboa, Portugal; 10 University of Liege, Institute of Botany, 4000 Liege, Belgium

## Abstract

Oceanic islands are of fundamental importance for the conservation of biodiversity because they exhibit high endemism rates coupled with fast extinction rates. Nowhere in Europe is this pattern more conspicuous than in the Macaronesian biogeographic region. A large network of protected areas within the region has been developed, but the question of whether these areas will still be climatically suitable for the globally threatened endemic element in the coming decades remains open. Here, we make predictions on the fate of the Macaronesian endemic bryophyte flora in the context of ongoing climate change. The potential distribution of 35 Macaronesian endemic bryophyte species was assessed under present and future climate conditions using an ensemble modelling approach. Projections of the models under different climate change scenarios predicted an average decrease of suitable areas of 62–87% per species and a significant elevational increase by 2070, so that even the commonest species were predicted to fit either the Vulnerable or Endangered IUCN categories. Complete extinctions were foreseen for six of the studied Macaronesian endemic species. Given the uncertainty regarding the capacity of endemic species to track areas of suitable climate within and outside the islands, active management associated to an effective monitoring program is suggested.

Oceanic islands are of fundamental importance for the conservation of biodiversity. Of the 34 biodiversity hotspots identified by Conservation International in 2005, 12 are or include island ecosystems. In fact, oceanic islands typically exhibit the attributes of the areas currently identified at hotspots in the biosphere, i.e. high endemism rates coupled with fast extinction rates^1^. The small population size and distribution range of island species, their unique biological features, especially the loss of their dispersal ability^2^, strong ecological specialization^1^,^3^, and their predicted low genetic diversity^1^, render them extremely vulnerable to environmental changes. Oceanic island floras are therefore among the most threatened worldwide^4^. Of the 80 documented plant extinctions in the last 400 years, 50 occurred on islands and more than 2000 endemic island taxa are currently thought to be on the verge of extinction^1^.

Nowhere in Europe is this pattern more conspicuous than in the oceanic islands of the Azores, Madeira and the Canaries that constitute, along with the archipelago of Cape Verde, the Macaronesian biogeographic region^1^,^5^. Plant biodiversity peaks in Macaronesia, with rates of endemism in angiosperms and in bryophytes of about 40% and 6.5%, respectively, so that the region is widely recognized as an outstanding biodiversity hotspot worldwide^1^. The flora of Macaronesia is, however, under considerable threat. Despite comprising less than 0.3% of Europe’s total land area, no fewer than 19% of habitats listed in Annex I of the Habitats Directive (European Directive 92/43 of 21 May 1992 on the conservation of natural habitats and of wild fauna and flora) and 28% of vascular plants in Annex II are endemic to Macaronesia. Among bryophytes, approximately 20% of the total bryophyte diversity has been included in Red Lists for the archipelagos of the Canaries and Madeira^6^,^7^, while 19% of the Azorean bryoflora is considered to be threatened^8^. Habitat loss due to human disturbance has been extensive and, for instance, it is estimated that only 12.5% of the unique Macaronesian laurel forest still remains^9^, this value decreasing to 5% in Azores^10^. A large network of protected areas and restoration actions within the region have been developed^11^, but the question of whether these areas will still be climatically suitable for the globally threatened endemic element in the next decades remains open.

In particular, laurel forests, including broadleaved laurel and ericaceous forest formations, represent the relicts of an evergreen humid forest community, which developed across the circum-Mediterranean region about 20 million years ago under climate conditions characterized by warm, wet summers^12^,^13^ (but see^14^). Since the onset of the Mediterranean climate, laurel forests only persisted in Macaronesia, where the oceanic environment buffered the climatic oscillations of the Pleistocene^9^, and where these forest ecosystems are closely associated with the elevational belt of orographic cloud formation^14^,^15^. The cloud layer results from the cooling of northeast trade winds blowing over the ocean surface and forced to ascend the mountain barriers of the islands, until they are trapped, at about 1,500 m a.s.l., by a layer of still, warm air, resulting in a typical thermal subsidence inversion^16^. This resident windward belt of clouds is of vital importance to the laurel forest because it creates a humid environment, which allows this ecological system to persist in the otherwise semi-arid climate of the Canary Islands^17^,^18^. This potentially makes the laurel forest ecosystem particularly sensitive to climatic change, which severely impacts the elevation and frequency of formation of the cloud belt^19^.

Specifically, the Canaries experienced a more pronounced global average temperature increase and precipitation decrease in the course of the last five decades than Madeira and the Azores^19^,^20^,^21^. Below the trade wind inversion, however, a significant increase in relative humidity and a decrease in diurnal temperatures were recorded throughout the last few decades^19^. In fact, an increase in low-level cloud cover and atmospheric moisture results in more solar radiation being reflected to space during the day while trapping more thermal infrared radiation emitted from the surface during the night. Climatic models projected into the future predict that the climatically suitable range for cloud laurel forests is likely to be extended downwards in a warmer world, since the limiting effects of thermal and hydric stress would be reduced at these elevations during the dry season^19^. In contrast, the upper level of the laurel forest ecosystem would be more frequently exposed to higher temperatures and intense radiation, as the cloud layer would cover this elevational range less often during the dry season. The reduced incidence of clouds at these elevations would not only threaten the upper limit of the laurel forest, but could have dramatic consequences for the island ecosystem functioning as these areas represent the few zones within the archipelago with a positive water balance throughout the year^18^.

Bryophytes are a group of spore-producing land plants, whose specific ecophysiological and biological features make them ideal candidates for investigating the impact of climate changes, leading Tuba *et al*.^22^ to describe them as ‘canaries in the coal mine’. In fact, their poikilohydric condition means that their water content is directly regulated by environmental humidity. Physiological activity, and hence growth, is restricted to periods of hydration^23^. In the absence of roots and a highly efficient internal water transport system, bryophytes hence depend primarily on surrounding water to sustain their needs^22^,^23^. Temperature is also a factor of prime importance in bryophyte physiology for regulating a suite of complementary mechanisms regarding growth and reproduction^24^. While bryophyte species are globally well equipped to grow at low temperature, all of the temperate and boreal species investigated by Furness and Grime^25^ died when kept continuously at 35 °C, with a majority of shoots already dying at >30 °C (see also^26^). These features are unlikely to evolve quickly in a changing environment, as the potential of bryophytes to become acclimatized to novel climatic conditions is, at least at the scale of a few decades, limited^26^. Indeed, even invasive species appear to lack the ability to expand their niche during the expansion process^27^. In the meantime, bryophytes are excellent dispersers, and recent evidence suggests that the European Atlantic fringe bryophyte flora assembled from Macaronesian ancestors since the end of the last glacial maximum, around 20,000 years ago^28^. This suggests that species currently restricted to Macaronesia might have the capacities to migrate towards western Europe provided that areas with suitable climatic conditions, among other ecological factors, will be available during the forthcoming decades.

Here, we use a robust implementation of species distribution models to investigate the fate of the endemic bryophyte flora of Macaronesia in the context of climate change. We specifically address the following questions: (i) to what extent will species distributions and elevational ranges be modified under different scenarios of climate change? In particular, we investigate whether western European areas will present climatic conditions that are compatible with the climatic niche of Macaronesian endemic species over the next decades; (ii) will the distributions of Macaronesian endemic bryophytes species be equally impacted across their range or are some archipelagos more threatened than others? (iii) Ultimately, to what extent will climate change cause a mismatch between future species distributions and the circumscription of protected areas as defined today?

## Results

**Figure 1:**
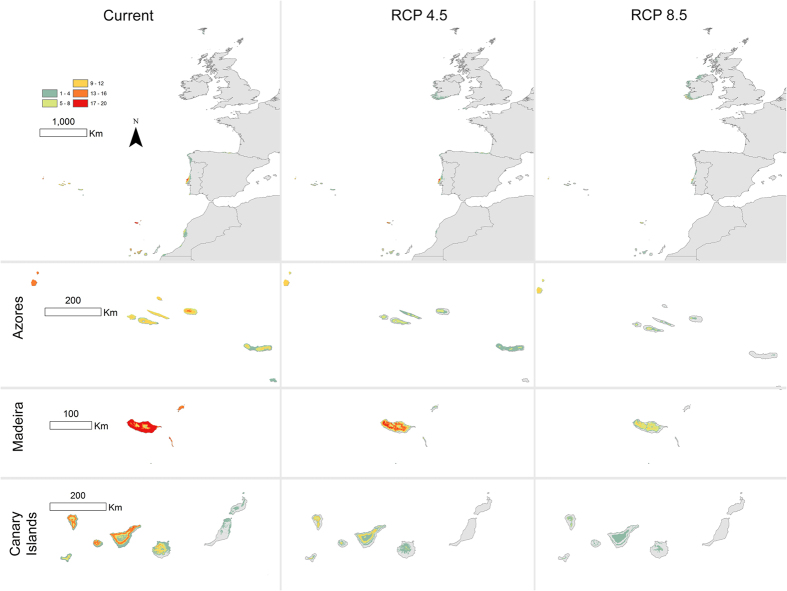
Modelled potential ranges of 35 Macaronesian endemic bryophytes species at present and 2070 under the contrasted climate scenarios defined by the RCP 4.5 and RCP 8.5 concentration pathways. The color scale represents the number of species for which macroclimatic conditions are defined as suitable for a given pixel. See Table S2 for results per archipelago. Maps were created using ArcGIS software by Esri (Environmental Systems Resource Institute, ArcGIS 10.0; www.esri.com).

The potential distribution area of all of the 35 investigated Macaronesian endemic bryophytes in 2070 is predicted to decrease substantially under the two climate scenarios of mitigated and strongly increased greenhouse gas emissions implemented here, which were defined by Representative Concentration Pathways (hereafter termed RCP) 4.5 and 8.5, respectively (representing two values of radiative forcing, in W/m^2^) (Figs 1 and S2). The average potential area (±SD) across species drops in 2070 to 38.3 ± 25.2% and 13.5 ± 13.6% of the extant potential area under the two scenarios defined by both the RCP 4.5 and RCP 8.5 concentration pathways, respectively. Under the RCP 8.5 scenario, six species (*Bryoxiphium madeirense*, *Echinodium setigerum*, *E*. *spinosum*, *Exsertotheca intermedia*, *Fissidens sublineaefolius*, and *Riccia atlantica*) are predicted to become totally extinct and the potential distribution area of another five species is predicted to reach less than 5% of the extant suitable area (Table 1).

Shifts in the extent of climatically suitable areas are paralleled by an elevational shift, as the vast majority of the investigated species exhibited a significant average increase of elevation between the present time and 2070 (Table 2). Non-significance of the average increase in elevation only occurred when species were predicted to disappear from some islands by 2070 (Table 2; see also Fig. S2), thereby decreasing the statistical power of the test.

**Figure 2:**
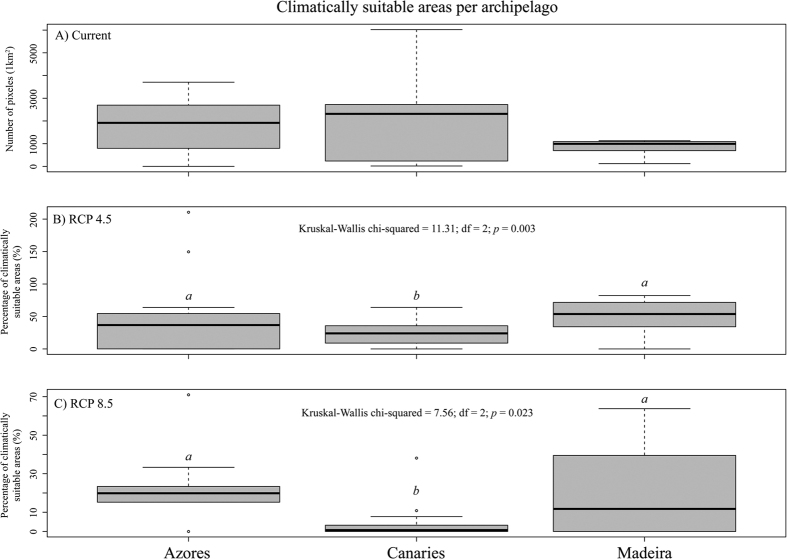
Percentage of climatically suitable areas in 2070 under the climate conditions defined by the RCP 4.5 (**B**) and RCP 8.5 (**C**) concentration pathways as compared to the present situation in both island and continental areas. The average of the total current climatically suitable areas (cells) per species is also provided (**A**). Boxes show the 25% and 75% quartiles, the horizontal line within the box is the median, while 10% and 90% percentiles are indicated by the whiskers and outliers by dots. Low-case letters denote homogenous groups according to pairwise comparisons using Tukey and Kramer (Nemenyi) test with Tukey-Dist approximation for independent samples at α = 0.05.

The decrease of macroclimatically suitable area by 2070 in the Canaries is significantly more severe (76.8 ± 18.3% and 96.2 ± 8.2% under the RCP 4.5 and RCP 8.5 respectively; see Fig. 2) than in the Azores (55.2 ± 54.4% and 80.5 ± 16.2%) and Madeira (50 ± 25.7% and 79.3 ± 23%) (Kruskal Wallis test, *p* = 0.003 and *p* = 0.023, respectively). Whereas Azores would lose five species, eight and nine taxa are predicted to be extinct in Madeira and the Canaries, respectively (Table S2; Fig. S2). Accordingly, the global circulation models employed here show that the increase in the maximum temperature of the warmest month and the precipitation decrease in the wettest month are substantially higher in the Canaries than in Madeira and, to a larger extent, the Azores (Table 3).

Under present conditions, 48.1 ± 17.4% of the potential distribution of the investigated species is included in protected areas (Table 4; see also Fig. S3). This proportion is expected to increase by 2070 to 64.5 ± 26.0% and 77.6 ± 19.1% under the two extreme scenarios defined by the RCP 4.5 and RCP 8.5 concentration pathways, respectively. Such a pattern is due to the higher decrease of climatically suitable areas outside than within protected areas (Fig. S3).

The projection of the models under present climatic conditions revealed that for 32 of the 35 Macaronesian bryophyte species investigated, climatically suitable conditions for their occurrence exist along a narrow fringe in the northwestern Iberian Peninsula (Fig. 1 and Fig. S2). This result represents, on average across species, 89.4 ± 106.3% of the potentially suitable area currently found in Macaronesia. For those 32 species, the RCP 4.5 projections predicted a decrease of suitable conditions on the continent in 2070, being completely unsuitable for six species. Thus, the potential continental area would represent 53.5 ± 95.9% of the currently suitable Macaronesia area on average across species. Under the RCP 8.5 scenario, climatic suitability for the investigated species on the continent decreases dramatically: the extent of the suitable area represents 16.7 ± 52.2% of the presently suitable Macaronesian area and the conditions become completely unsuitable for 17 species (see Table 1 and Fig. S2).

## Discussion

Projections of the macroclimatic niche of the 35 Macaronesian endemic bryophyte species under two different climate change scenarios of greenhouse gas concentration pathways at the scale of Macaronesia point to a substantial decrease of the climatic suitability by 2070, with a potentially suitable area that only represents between ca. 38% and 13% of the extant range depending on the scenario used (RCP 4.5 and RCP 8.5, respectively). The substantial decrease in suitable areas by 2070 was paralleled by a significant increase in the average elevation range, as if species compensate for the temperature increase and precipitation decrease by an elevational shift.

Such a predicted increase in the elevation range of Macaronesian endemic bryophyte species, 87% of which are restricted to the laurel forest^29^, is consistent with a growing body of evidence pointing to climate-driven distribution shifts towards higher elevations, including tropical areas^30^,^31^. However, our results do not fit with models predicting the downward shift of the macroclimatically suitable area for this vegetation belt^19^. As opposed to those of Sperling *et al*.^19^, our predictive models did not include humidity variables, such as the mean moisture index of the coldest quarter into account. Nevertheless, this variable was highly correlated with the precipitation of the warmest quarter that was employed here (*R* = 0.90; *p* < 0.001), suggesting that, at a macroclimatic scale across Macaronesia, vertical and horizontal precipitations are strongly correlated. Although the incongruence with the predictions of Sperling *et al*.^19^ could be explained by differences in the circulation models used (second versus fifth IPCC Assessment Report in Sperling *et al*.^19^ and the present study, respectively), such a difference suggests that the response of endemic Macaronesian bryophytes to future climate change could be decoupled from that of their current main ecosystem, the laurel forest.

Given the comparatively coarse spatial grain of our study (approximately 1–km^2^ grid resolution), the hypothesis that some species will persist in small microhabitats such as ravines, where humidity can be higher than in the surrounding environment^32^, cannot be rejected. Nevertheless, our results point to a substantial decline of the macroclimatically suitable area for Macaronesian endemic bryophytes. Thus, the present study suggests that, within the next few decades, even the commonest species such as *Homalothecium mandonii* and *Exsertotheca intermedia*, might substantially change of conservation status and fit either the Vulnerable (30% range reduction) or Endangered (50% range reduction) IUCN categories defined for oceanic island species^7^. These predictions are even more dramatic at the scale of the Canary Islands, where a decrease of no less than 77–96% is predicted by 2070. As compared to extant levels of threat, with seven critically endangered and 20 endangered species in the Canary Islands^7^ and five critically endangered and 22 endangered in Madeira^6^, our findings point to an extreme increase of the extinction risk within the next decades. Indeed, complete extinctions are predicted for eight Madeiran and nine Canarian species under the RCP 8.5 climatic scenario and a further three and one species, respectively, are expected to be near-extinct with less than 1% of their currently suitable area remaining.

The predicted extinction of approximately 17.1% of the Macaronesian endemic bryophyte species by 2070 is higher than the average of 7.9% of extinction due to climate change when different taxa (including birds, reptiles, amphibians, invertebrates, mammals, fish and plants) and biogeographic regions were considered^33^ and to the 13.9% predicted for endemic species worldwide^33^. This result suggests that Macaronesian endemic bryophytes, which are largely restricted to long-term macroclimatically stable ecosystems such as the laurel forest^29^, will be the first, along with other highly sensitive taxa like amphibians and reptiles^33^, to disappear in a warmer world. A similar impact of climate change was, however, predicted for the genus *Sideritis* (Lamiaceae), with a decrease in distribution area for most species and a high risk of extinction for 1–8 of the 23 species present in Macaronesia by 2080^34^, pointing to the short-term threat on Macaronesian biodiversity as a whole.

Our predictions further suggest that climate change will not affect the Macaronesian bryoflora homogeneously across its range. Thus, predictions for the decrease of the macroclimatically suitable area by 2070 are substantially more severe in the Canaries (77–96% on average depending on the investigate climate change scenarios) than in the Azores (55.2–80.5%) and Madeira (50–79.3%). Indeed, the increase in the maximum temperature of the warmest month and the decrease in the precipitation of the wettest month by 2070 are expected to be substantially higher in the Canaries than in Madeira and, to a larger extent, Azores. Given the poikilohydric condition of bryophytes and the narrow ecophysiological niche of laurel forest Macaronesian endemic bryophytes^35^, the global tendency towards warmer and drier climates in the Canaries than in the Azores and Madeira is indeed expected to affect the endemic bryophyte floristic element more severely in the former than in the latter archipelagos. This points to the need for a higher conservation effort in the Canaries to maintain the original composition and structure of the native ecosystems, particularly in laurel forest remnants, but also to the high importance of Madeira and the Azores as refugia for the conservation of Macaronesian endemic bryophytes under the global warming. The latter means that additional efforts to manage properly protected areas and restore the natural ecosystems in these two northern archipelagos should be also implemented.

Furthermore, whereas areas of suitable climate conditions are predicted to markedly decrease across the distribution range of Macaronesian endemic bryophyte species, the proportion of protected area with suitable conditions is paradoxically predicted to exhibit the reverse trend, with an increase from the extant average level of 47.0 ± 18.1% to 63.6 ± 25.5 and 74.3 ± 33.5% of the suitable area in 2070 under the two extremes defined by the RCP 4.5 and RCP 8.5 greenhouse gas concentration pathways. Since these proportions reflect the number of suitable pixels within protected areas divided by the total number of suitable pixels across the entire Macaronesian range at present time and in 2070 (see Methods), the present study suggests that climatically suitable areas will increasingly be restricted to protected areas during the next decades. Therefore, our results emphasize the importance of protected areas for the conservation of Macaronesian biodiversity in the future.

Finally, our results identify a narrow range along the western fringe of the Iberian Peninsula as climatically suitable for Macaronesian endemic bryophytes. These Atlantic continental areas indeed exhibit physionomically similar evergreen broadleaved (e.g. the tree genera *Prunus* and *Laurus*) forests to those found in Macaronesia, and they host a conspicuous Atlantic element shared in part (e.g. *Ulota calvescens, Sematophyllum substrumulosum*) or exclusively (e.g. *Tetrastichium* spp, *Neckera cephalonica*) with Macaronesia. The climatic suitability of those continental regions, along with the predicted upward shift of all the species distributions investigated to areas, where the laurel forest does not occur, raise the question of whether Macaronesian endemic bryophytes display the dispersal and establishment capacities to colonize new suitable areas and adapt to habitat conditions outside of the laurel forest in the forthcoming decades, both within islands and/or neighboring continental areas. Bryophyte species appear to exhibit a large ability to colonize secondary habitats following human disturbance, provided that suitable ecological and climatic conditions exist^36^,^37^. In particular, many of the investigated endemic species, although largely restricted to the laurel forest, can be found on various substrates outside of the laurel forest itself, even in suboptimal disturbed habitats or in very specialized habitats with constant humidity as lava tube cave entrances or pit caves. For instance, a rich suite of species that are primarily found in the ancient laurel forest across the Canaries was able to exceptionally colonize *Pinus radiata* plantations in areas characterized by extremely high frequency of mountain fogs (J. Patiño, pers. obs.). In Azores, a few colonies of *Echinodium renauldii* are known to occupy disturbed forest patches^38^ or lava tube entrances (R. Gabriel and P Borges, pers. obs.). Other species such as *Ptychomitrium nigrescens*, which are characteristic for the laurel forest altitudinal belt in the Canaries, can also be found at low elevation on islands that, like El Hierro, display the adequate climatic conditions for these species. Furthermore, recent phylogeographic evidence suggests that formerly endemic Macaronesian species have colonized several times the western Atlantic fringe of Europe since the last glacial maximum^28^. Within the endemic element of the Macaronesian bryophyte flora, the strong genetic structure observed at small scales^39^,^40^,^41^ and significant shifts in the expression of mating systems and associated dispersal life-history traits^2^ point, however, to dispersal limitations. These observations raise substantial concerns on the ability of Macaronesian bryophyte species to migrate to macroclimatically suitable areas on the continent as a response to the dramatic decrease of their suitable areas on the islands during the next decades. The current direction of the trade winds (i.e. from northeast to southwest) further accentuates the geographic isolation of the Macaronesian wind-dispersed flora, which creates another dispersal barrier for continental colonization^5^.

Altogether, our predictions suggest that, while Macaronesia appears to have been a climatic refugium for species that either went extinct on continents^40^,^42^,^43^ or back-colonized continental areas during postglacial periods^39^,^44^, and has even been a source of novel biodiversity for neighbouring continental regions^28^,^45^, its role as a historical sanctuary for the bryophyte flora might be severely threatened in the ongoing context of global warming. This is particularly true if long-term meteorological station records across Macaronesia, indicating a significant increase in temperature and decrease in precipitation over the last decades^20^,^21^,^46^, are taken into account. Many island endemics have a small distribution and are confined to specific climatic and edaphic conditions, exhibiting narrow realised ecological niches^47^, as it is the case of eight species that are only known from one to a few localities (i.e. *Echinodium setigerum*, *Fissidens azoricus*, *Fissidens nobreganus*, *Frullania sergiae*, *Hedenasiastrum percurrens*, *Orthotrichum handiense*, *Radula jonesii*, *Riccia atlantica*). Endemic bryophyte species are, however, not necessarily narrowly distributed specialists. Rather, some species, and in particular, large pleurocarpous mosses, dominate the ground layer in certain habitats. For example, *Homalothecium mandonii* thrives on the ground in pine woodland and xeric shurb vegetations, while *Andoa berthelotiana* and *Exsertotheca intermedia* can form large pendent mats on the branches and trunks in the laurel forest. Since the locally important contribution of large carpets of terrestrial and epiphyte pleurocarpous moss species to the biomass of temperate and tropical forests, respectively, these climatic-driven distribution changes might potentially have severe functional consequences in terms of water storage, nutrient cycling and availability of microhabitats for other organisms^22^,^26^.

Given the serious threat due to anthropogenic pressure and derived problems such as cattle, fires, invasiveness and deforestation^4^, and given the uncertainty regarding the capacity of endemic species to track areas of suitable conditions following climate change both within and outside the Macaronesian islands, we suggest that the *ex-situ* conservation of at least the rarest species known from a single to a few localities would be advisable. Bryophytes are well suited for cryopreservation and such techniques would be applicable for the long-term storage of diaspores of highly threatened species^48^ for their subsequent reintroduction under favourable environmental conditions^6^,^7^. In this context, ongoing research on the spatial genetic structure of rare Macaronesian endemic bryophyte species (e.g.^40^) will help establishing the bases for sound population translocation actions.

## Methods

### Data sources

Information on species distributions was collected from the databases maintained by us for each archipelago based on verified herbarium records, thorough literature reviews and actual field observations (Table S1). In total, 2091 occurrences were obtained. Species distributions were geo-referenced using a 0.0083 decimal degrees grid resolution (approximately 1–km^2^, according to the pixel resolution of the environmental variables data). To avoid sampling bias^49^, only points that were separated by at least 0.0083 decimal degrees from each other (i.e. matching the resolution of the climatic data) were eventually retained.

The Macaronesian endemic bryophyte flora includes 47 species (6.5% of the bryoflora), 30 mosses and 17 liverworts^50^,^51^,^52^,^53^,^54^, which are largely restricted (87%) to the laurel forest^29^. Modeling the distribution of narrow endemic species raises the issue of low sample sizes^55^. In fact, van Proosdij *et al*.^56^ suggested that the minimum sample size for species distribution models ranges between 3 and 13 occurrences depending on the proportion of the study area occupied by a given species and the specific ecological features of the targeted study area. Here, we used an intermediate threshold and focused on 23 moss and 12 liverwort species with more than ten records^56^. Therefore, the following species were not considered in the present study: *Fissidens azoricus* on Flores, *Trematodon perssoniorum* on Sao Miguel and *Thamnobryum rudolphianum* on Faial, all occurring in the Azorean archipelago; *Frullania sergiae*, *Fissidens nobreganus* and *Nobregaea latinervis* on Madeiran archipelago; and *Orthotrichum handiense* on Fuerteventura and *Aloina humilis*, *Riccia teneriffae* on Tenerife, all from the Canaries. Other Macaronesian endemics such as *Cololejeunea maderensis, Radula jonesii* and *Lejeunea canariensis* were neither considered. The lack of detailed information for the Cape Verde endemic bryophyte species (in total six taxa; see^57^) also precluded their inclusion in the present study. The nomenclature follows Ros *et al*.^50^ for liverworts and Ros *et al*.^58^ for mosses (see Table S1).

The definition of the geographic background (i.e. the extent of the study area defined to calibrate the model) is of prime importance, as it may not only affect the calibration of the models, but most importantly their transferability in space and time^59^. Acevedo *et al*.^59^ suggested that the geographic background should not only reflect the extant, but also the potentially occupied range in the past. In Macaronesia, Engler^12^, subsequently followed by Sunding^13^ among others, proposed that the distinctive endemic element of the Macaronesian flora was, for the most part, a relict of a formerly widespread subtropical flora that covered southern Europe and North Africa during the Tertiary and vanished from northern Africa and the Mediterranean with the onset of cold climates since the end of the Tertiary^9^,^60^. Whether such a theory applies to bryophytes has been challenged^61^, but evidence from molecular dating analyses suggests that, in some instances, Macaronesian endemic bryophyte species originated much before the islands actually emerged, unambiguously pointing to their palaeo-endemic origin^40^,^42^,^43^,^45^. For these reasons, the geographic background employed in the present study encompassed a much larger area than the extant distribution of the studied species and also included large areas of Europe, North Africa and the southernmost Macaronesian archipelago of Cape Verde (see Fig. S1).

Nineteen bioclimatic variables were employed as environmental predictors and sampled at a resolution of 30 arc-seconds (approximately one km^2^) from WorldClim 1.4^62^. WorldClim does not include the large panel of variables available from other sources such as CliMond^63^, but was selected because it implements the most recent global climate model (GCM) data from CMIP5 (IPPC Fifth Assessment) for future conditions. To ensure that key variables, such as air humidity, were not discarded in the present study, we computed the correlation (Pearson correlation) between the variables of temperature and precipitation selected for the present study and all of the other variables available in CliMond at the 5 km^2^ resolution scale under present conditions across Macaronesia.

Finer-scaled data would better capture the local effects of topographic complexity observed in some of the Macaronesian islands, but are, except for the Azores and Madeira^64^, not available across the entire geographic background used here. Decreasing the grain of the study would further require the implementation of other variables of land use, soil condition, and biotic interactions, whose importance increases when the geographic scale of the study decreases, whereas the present study aims, given its large geographical extent, at assessing global patterns driven by macroclimatic conditions.

Following Pearson *et al*.^55^, who included 20 variables to model the distribution of species with up to five presence records, we decided to consider several variables at the initial stage of model building, to keep the potential to describe a global climate setting rather than focusing on one or a few variables. However, we decreased the number of variables to be included in the model by eliminating one of the variables in each pair with a Pearson correlation value >0.8 based on a random sampling of 10,000 points over the geographic background, keeping the variables that are the most relevant in explaining bryophyte distributions^22^,^24^,^25^. After elimination of the redundant variables, five variables were included in the model as follows: BIO5 (maximum temperature of warmest month), BIO6 (minimum temperature of coldest month), BIO13 (precipitation of wettest month), BIO14 (precipitation of driest month), and BIO18 (precipitation of warmest quarter). Inclusion of the maximum temperature of warmest month is justified by the high relevance of this factor on mortality rates in temperate bryophytes^25^, while precipitation levels at different temperatures appear as a crucial factor given the poikilohydric conditions of bryophytes, controlling their growth rates^65^ (for a review see^26^).

### Data analyses

An ensemble model of three different techniques as implemented by BIOMOD 2.0^66^ was used to model the distribution of the 35 Macaronesian endemic bryophytes species selected. The techniques included BIOCLIM^67^, MaxEnt^68^, and Random Forests^69^, which were recommended for datasets with small sample size^55^.

For each technique, presences and pseudo-absences used to calibrate the model under current climatic conditions were weighted such as to ensure neutral (0.5) prevalence. The performance of the models was assessed by randomly splitting ten times the data, keeping 70% of them to generate the models and employing the remaining 30% to evaluate their performance based on the AUC criterion (area under the ROC curve) and TSS statistic (true skill statistic). After elimination of all models with an AUC < 0.8 or TSS < 0.7, we generated for each species a consensus model, in which the contribution of each individual technique was proportional to its AUC.

The consensus model was then projected onto the future climate conditions. Future projections were derived using two different Global Climate Models recommended for Europe and Mediterranean climates^70^, namely MPI-ESM-LR (Max-Planck-Institut für Meteorologie in Germany) and HadGEM2-ES (Hadley Centre for Climate Prediction and Research in UK) under two RCP (representative concentration pathways) 4.5 and 8.5^71^ proposed by the Intergovernmental Panel on Climate Change in the Fifth Assessment report. The RCP 4.5 is a stabilization scenario where total radiative forcing is stabilized before 2100 by employment of a range of technologies and strategies for reducing greenhouse gas emissions’, whereas the ‘RCP 8.5 is characterized by increasing greenhouse gas emissions over time representative for scenarios in the literature leading to high greenhouse gas concentration levels’^71^. Maps of the potential climate suitability for each species were finally generated. For that purpose, the continuous suitability index was transformed into a binary presence/absence model^72^,^73^, using a 5% commission error adjustment.

Projecting the models in areas or time periods where climate conditions are not analogous to those prevailing in the area where the model was built can, however, return unreliable results^74^. We therefore identified which areas of Macaronesia will exhibit analogous climates in 2070 as compared with present time, and which areas of the continental areas currently exhibit, and will exhibit in 2070 analogous climates to those currently observed in Macaronesia (Fig. S3) using multivariate environmental similarity surfaces (MESS)^74^. MESS measures the similarity of any given pixel in the continent to the full set of selected pixels in Macaronesia with respect to the chosen predictor variables. A pixel with a positive value indicates that it falls within the range of environmental values present on islands, while a pixel with a negative value indicates that at least one variable has a value that is outside of the range of environmental values present on islands. Any area identified as suitable using the models, but falling outside of the area of analogous climates, was therefore not considered.

To determine whether the species elevational ranges will be modified by climate change, we computed the average elevation predicted as macroclimatically suitable for each species across the Macaronesian islands. We then used a pairwise Student’s t test to assess whether the mean elevation will significantly vary between the present time and 2070 under the two RCP across islands. Kruskal–Wallis tests were carried out in order to test the differences in percentage of climatically suitable areas in 2070 under the climate conditions defined by the concentration pathways RCP 4.5 and RCP 8.5 as compared to the present situation, among archipelagos. *Post hoc* (Nemenyi) test were then carried out, using the ‘posthoc.kruskal.nemenyi.test’ function in the Comparison of Mean Ranks Package package^75^ (PMCMR). All statistical analyses and species distribution models were carried out in R (version 3.2.3, Development Core Team, http://cran.r-project.org).

Any area of the Macaronesian archipelagos of Azores, the Canaries and Madeira defined as National Parks, Nature Reserves or Natura 2000 sites was considered as protected areas. Protected area polygons were obtained from the World Database on Protected Areas (WDPA: IUCN & UNEP-WCMC, 2013), with a total of 545 protected area polygons across the Macaronesian region. The proportion of macroclimatically suitable areas within protected areas was computed as the number of macroclimatically suitable pixels within protected areas divided by the total number of macroclimatically suitable pixels, at present time and in 2070 under the RCP 4.5 and 8.5, respectively. Following a conservative approach, we considered that a climatically suitable pixel was inside a natural protected area, if at least 5% of that pixel was included within a protected area.

## Additional Information

**How to cite this article**: Patiño, J. *et al*. Climate threat on the Macaronesian endemic bryophyte flora. *Sci. Rep.* **6**, 29156; doi: 10.1038/srep29156 (2016).

## Supplementary Material

Supplementary Information

## Figures and Tables

**Table 1:** Number of 1–km^2^ climatically suitable pixels for each of 35 investigated Macaronesian endemic bryophyte species in Macaronesia (nMac), and western Europe and northwestern Africa (nCONT) under present climate conditions, and in 2070 under the climate conditions defined by the RCP 4.5 and RCP 8.5 concentration pathways.

	PRESENT		RCP 4.5		RCP 8.5	
	nMAC	nCONT	nMAC	nCONT	nMAC	nCONT
*Alophosia azorica*	2698	766	1473 (54.6)	945 (123.4)	451 (16.7)	0 (0)
*Andoa berthelotiana*	3706	763	2077 (56)	580 (76)	879 (23.7)	0 (0)
*Bazzania azorica*	2066	1681	572 (27.7)	13 (0.8)	522 (25.3)	0 (0)
*Breutelia azorica*	2093	2134	856 (40.9)	170 (8)	428 (20.4)	1 (0)
*Bryoxyphium madeirense*	1157	321	349 (30.2)	1046 (325.9)	0 (0)	0 (0)
*Calypogeia azorica*	2353	2283	943 (40.1)	252 (11)	469 (19.9)	0 (0)
*Cheilolejeunea cedercreutzii*	694	189	198 (28.5)	0 (0)	161 (23.2)	0 (0)
*Cololejeunea schaeferi*	2406	358	672 (27.9)	1033 (288.5)	14 (0.6)	107 (29.9)
*Cryptoleptodon longisetus*	3395	809	1465 (43.2)	1799 (222.4)	336 (9.9)	361 (44.6)
*Echinodium renauldii*	2783	0	1046 (37.6)	0 (0)	651 (23.4)	0 (0)
*Echinodium setigerum*	749	672	304 (40.6)	2740 (407.7)	0 (0)	0 (0)
*Echinodium spinosum*	1034	536	303 (29.3)	692 (129.1)	0 (0)	0 (0)
*Exsertotheca intermedia*	8193	7905	2086 (25.5)	2207 (27.9)	0 (0)	0 (0)
*Fissidens coacervatus*	3991	3724	2202 (55.2)	2880 (77.3)	658 (16.5)	985 (26.5)
*Fissidens nobreganus*	1089	2473	474 (43.5)	429 (17.3)	1 (0.1)	0 (0)
*Fissidens sublineaefolius*	1344	4518	512 (38.1)	1025 (22.7)	0 (0)	0 (0)
*Frullania polysticta*	5073	9522	2283 (45)	2438 (25.6)	890 (17.5)	789 (8.3)
*Grimmia curviseta*	679	251	61 (9)	0 (0)	9 (1.3)	0 (0)
*Hedenasiastrum percurrens*	706	2133	235 (33.3)	21 (1)	8 (1.1)	0 (0)
*Heteroscyphus denticulatus*	6682	4470	1050 (15.7)	2747 (61.5)	369 (5.5)	461 (10.3)
*Homalothecium mandonii*	6288	6316	4191 (66.7)	3397 (53.8)	2526 (40.2)	852 (13.5)
*Isothecium prolixum*	2337	691	842 (36)	0 (0)	454 (19.4)	12 (1.7)
*Leptoscyphus azoricus*	802	276	171 (21.3)	0 (0)	122 (15.2)	0 (0)
*Leucodon canariensis*	4836	7661	1314 (27.2)	1513 (19.7)	451 (9.3)	357 (4.7)
*Leucodon treleasei*	1768	0	2644 (149.5)	0 (0)	830 (46.9)	0 (0)
*Pelekium atlanticum*	3377	3949	1360 (40.3)	1049 (26.6)	248 (7.3)	333 (8.4)
*Plagiochila maderensis*	3079	3465	1455 (47.3)	1537 (44.4)	252 (8.2)	313 (9)
*Porella inaequalis*	1368	6591	950 (69.4)	6027 (91.4)	733 (53.6)	3849 (58.4)
*Radula wichurae*	3506	64	2239 (63.9)	555 (867.2)	688 (19.6)	0 (0)
*Rhynchostegiella bourgaeana*	2568	91	241 (9.4)	0 (0)	21 (0.8)	4 (4.4)
*Rhynchostegiella macilenta*	3435	1968	1723 (50.2)	2199 (111.7)	757 (22)	986 (50.1)
*Rhynchostegiella trichophylla*	3445	260	905 (26.3)	140 (53.8)	201 (5.8)	375 (144.2)
*Riccia atlantica*	174	0	2 (1.1)	0 (0)	0 (0)	0 (0)
*Telaranea azorica*	796	152	309 (38.8)	0 (0)	162 (20.4)	0 (0)
*Tortella limbata*	2997	2789	282 (9.4)	721 (25.9)	276 (9.2)	174 (6.2)

The numbers between parentheses represent the percentage of climatically suitable areas as compared to the present situation in both island and continental areas.

**Table 2:** Variation in the predicted potential average elevational range (±SD) of Macaronesian endemic bryophyte species per island (n = 19) between the present time and 2070 under the climate conditions defined by the RCP 4.5 and RCP 8.5 concentration pathways.

**Species**	**Present**	**RCP 4.5**	**RCP 8.5**
*Alophosia azorica*	323 ± 142	453 ± 182**	639 ± 286*
*Andoa berthelotiana*	269 ± 99	400 ± 169*	483 ± 237**
*Bazzania azorica*	368 ± 178	567 ± 296*	603 ± 282*
*Breutelia azorica*	380 ± 165	546 ± 231**	651 ± 320*
*Bryoxiphium madeirense*	744 ± 698	2223 ± 1015	Extinct
*Calypogeia azorica*	350 ± 161	515 ± 220**	694 ± 327**
*Cheilolejeunea cedercreutzii*	573 ± 254	742 ± 340*	716 ± 436
*Cololejeunea schaeferi*	613 ± 402	1247 ± 210**	1364 ± 335
*Cryptoleptodon longisetus*	510 ± 366	1118 ± 380**	1322 ± 513**
*Echinodium renauldii*	263 ± 100	451 ± 183*	586 ± 242**
*Echinodium setigerum*	569 ± 213	1834 ± 1102	Extinct
*Echinodium spimosum*	612 ± 246	1143 ± 1531*	Extinct
*Exsertotheca intermedia*	255 ± 299	1363 ± 291**	Extinct
*Fissidens coacervatus*	502 ± 448	885 ± 431*	1355 ± 252***
*Fissidens nobreganus*	1190 ± 1069	1094	Extinct
*Fissidens sublinaefolius*	383 ± 355	992	Extinct
*Frullania polysticta*	474 ± 374	965 ± 466***	1454 ± 268**
*Grimmia curviseta*	1851 ± 157	2521 ± 557	Extinct
*Hedenasiastrum percurrens*	1224 ± 431	981	Extinct
*Heteroscyphus denticulatus*	436 ± 415	1193 ± 383**	2118 ± 1434
*Isothecium prolixum*	370 ± 156	578 ± 240*	689 ± 297**
*Leptoscyphus azoricus*	559 ± 247	723 ± 449	766 ± 421*
*Leucodon canariensis*	426 ± 399	1292 ± 227**	1493 ± 428
*Leucodon treleasei*	113 ± 50	203 ± 84**	766 ± 158***
*Homalothecium mandonii*	434 ± 356	709 ± 455**	970 ± 497*
*Pelekium atlanticum*	580 ± 462	1213 ± 186**	1291
*Plagiochila madeirensis*	577 ± 461	1242 ± 172**	1287
*Porella inaequalis*	731 ± 961	1062 ± 909	1482 ± 1514
*Radula wichurae*	262 ± 107	421 ± 175*	615 ± 274*
*Rhynchostegiella bourgeana*	367 ± 314	1002 ± 68***	1466 ± 112**
*Rhynchostegiella macilenta*	536 ± 431	987 ± 357**	1346 ± 297*
*Rhynchostegiella trichophylla*	524 ± 359	1222 ± 87***	1433 ± 86***
*Riccia atlantica*	29 ± 30	651	Extinct
*Telaranea azorica*	550 ± 248	747 ± 321**	796 ± 391*
*Tortella limbata*	537 ± 536	2254 ± 121	1480 ± 1254

*, ** and *** indicate the significance of the average difference in elevation between the present time and 2070 at the 0.05, 0.01 and 0.001 significance levels, respectively.

**Table 3:** Variation in the average (±SD) maximum temperature of the warmest month and the average precipitation of the wettest month across islands of the Canarian, Madeiran, and Azorean archipelagos at present time (data for the period 1950–2000) and in 2070 under the climate conditions defined by the RCP 4.5 and RCP 8.5 concentration pathways.

	**Present**	**RCP 4.5**	**RCP 8.5**	
Maximum temperature of the warmest month				
Canaries	26.7 ± 0.8	28.7 ± 0.8	29.9 ± 0.7	
Madeira	24.1 ± 0.5	26.5 ± 1.2	27.6 ± 1.2	
Azores	24.1 ± 0.5	26.9 ± 0.5	28.2 ± 0.5	
Precipitation of the wettest month (mm)				
Canaries	51.9 ± 24.1	43.3 ± 22.9	40.4 ± 20.2	
Madeira	82 ± 16	69.5 ± 15.6	54.3 ± 10.8	
Azores	150.8 ± 33.9	143.1 ± 29.5	138.2 ± 33.3	

**Table 4:** Variation in the proportion of the predicted climatically suitable area of Macaronesian endemic bryophyte species that is included within a legally protected area for the present and in 2070 under the climate conditions defined by the concentration pathways RCP 4.5 and RCP 8.5.

	**PRESENT**	**RCP 4.5**	**RCP 8.5**
*Alophosia azorica*	29.1	42.0	55.0
*Andoa berthelotiana*	24.4	32.4	50.7
*Bazzania azorica*	32.5	57.9	56.5
*Breutelia azorica*	32.0	57.1	55.1
*Bryoxyphium madeirense*	60.3	97.4	Extinct
*Calypogeia azorica*	29.7	50.2	53.7
*Cheilolejeunea cedercreutzii*	58.6	42.9	60.9
*Cololejeunea schaeferi*	47.0	89.0	100.0
*Cryptoleptodon longisetus*	51.6	82.1	92.3
*Echinodium renauldii*	21.5	43.2	53.8
*Echinodium setigerum*	58.7	95.4	Extinct
*Echinodium spinosum*	61.7	96.4	Extinct
*Exsertotheca intermedia*	47.3	84.7	Extinct
*Fissidens coacervatus*	54.7	67.1	85.7
*Fissidens nobreganus*	65.9	92.2	100.0
*Fissidens sublineaefolius*	54.4	88.5	Extinct
*Frullania polysticta*	58.2	74.9	90.4
*Grimmia curviseta*	100.0	100.0	100.0
*Hedenasiastrum percurrens*	77.8	100.0	100.0
*Heteroscyphus denticulatus*	43.6	82.2	94.9
*Homalothecium mandonii*	57.3	64.4	77.1
*Isothecium prolixum*	30.4	56.2	56.2
*Leptoscyphus azoricus*	56.7	36.8	69.7
*Leucodon canariensis*	50.3	86.5	96.9
*Leucodon treleasei*	15.3	16.1	70.7
*Pelekium atlanticum*	55.7	80.4	100.0
*Plagiochila maderensis*	55.0	82.4	100.0
*Porella inaequalis*	65.0	68.0	77.8
*Radula wichurae*	24.3	30.1	58.4
*Rhynchostegiella bourgaeana*	46.2	50.2	100.0
*Rhynchostegiella macilenta*	52.0	68.1	91.9
*Rhynchostegiella trichophylla*	51.2	70.1	96.5
*Riccia atlantica*	26.4	0.0	Extinct
*Telaranea azorica*	51.9	51.5	61.1
*Tortella limbata*	63.8	24.1	47.8
