# Supplementary material for: Climate threat on the Macaronesian endemic bryophyte flora
Source: Sci Rep. 2016 Jul 5;6:29156. doi: 10.1038/srep29156 (PMC4932530; doi:10.1038/srep29156)

Supplementary Information

Climate threat on the Macaronesian endemic bryophyte flora

Jairo Patiño[1,2,3,4], Ruben G. Mateo[2], Florian Zanatta[1], Adrien Marquet[1], Silvia C. Aranda[5], Paulo A. V. Borges[3], Gerard Dirkse[6], Rosalina Gabriel[3] Juana M. Gonzalez-Mancebo[4], Antoine Guisan[2,7], Jesús Muñoz[8], Manuela Sim-Sim[9], Alain Vanderpoorten[1,3]

[1]Island Ecology and Evolution Research Group, Instituto de Productos Naturales y Agrobiología (IPNA-CSIC), Tenerife, Canary Islands, Spain. [2]Department of Ecology and Evolution, University of Lausanne, Lausanne, Switzerland. [3]Centre for Ecology, Evolution and Environmental Changes (cE3c)/Azorean Biodiversity Group & University of the Azores, Angra do Heroísmo, Azores, Portugal. [4]Department of Plant Biology, University of La Laguna, Tenerife, Spain. [5]Museo Nacional de Ciencias Naturales (CSIC), 28006 Madrid, Spain. [6]Natuurmuseum Nijmegen, Nijmegen, The Netherlands. [7]Institute of Earth Surface Dynamics, University of Lausanne, Lausanne, Switzerland. [8]Real Jardín Botánico (CSIC), Madrid, Spain. [9]Centre for Ecology, Evolution and Environmental Changes (cE3c), Universidade de Lisboa, Faculdade de Ciências de Lisboa, Lisboa and Museu Nacional de História Natural e da Ciência, Jardim Botânico, Lisboa, Portugal.

Correspondence and requests for materials should be addressed to J.P. (email: jpatino.llorente@gmail.com).

Table S1. List of herbarium vouchers, field observations and references used to compile the dataset for each endemic bryophyte species for the archipelagos of Madeira and the Canary Islands. The information for Azores is included in Appendix S1.

Andoa berthelotiana **(Mont.) Ochyra**

Madeira

Vouchers: LISU 149059; LISU 149126; LISU 149127; LISU 149128; LISU 149129; LISU 149130; LISU 161970; LISU 161971; LISU 161972; LISU 161977; LISU 161978; LISU 161979; LISU 238835; LISU 240725; LISU 240775; LISU 241083; LISU 241430; LISU 241827; LISU 242371; LISU 244232; LISU 253228; LISU 257631; LISU 257649; LISU 257651; LISU 257704; LISU 257711; LISU 257940; LISU 257972; LISU 257974; LISU 258055; LISU 258151; LISU 258195; LISU 258217; LISU 258265; LISU 258425; LISU 258435; LISU 258485; LISU 258492; LISU 258604; LISU 258610; LISU 258642; LISU 258681; LISU 258774; LISU 258782; LISU 258816; LISU 258889; LISU 258909; LISU 258958; LISU 259042; LISU 259070; LISU 259089; LISU 259091; LISU 259164; LISU 259198; LISU 259285; LISU 259300; LISU 259361; LISU 259410; LISU 259516; LISU 259516; LISU 259536; LISU 259536; LISU 259614; LISU 259626; LISU 259643; LISU 259661; LISU 259699; LISU 259700; LISU 259743; LISU 259746; LISU 259852; LISU 259923; LISU 259951; LISU 259986; LISU 260000; LISU 260013; LISU 260027; LISU 260095; LISU 260106; LISU 260115; LISU 260119; LISU 260143; LISU 260176; LISU 260230; LISU 260315; LISU 260341; LISU 260761; LISU 260767; LISU 260780; LISE 6965;LISE 7130; SB8339; SB8308; SB8314; SB8340; SB8341; SB8347; SB8366; SB9599; MADS1378; SB43205; SB8333; SB8378; MADJ8330; MADJ6826; SB8315; SB8380; SB8321; MADS1379; MADS1364; SB8376; SB8329; SB8330

The Canaries

Vouchers: GMD 20036; GMD 20031; GMD 20034; GMD 20038; GMD 20042; GMD 20028; GMD 20037; GMD 4151; GMD 3467; GMD 3334; GMD 3700; GMD 5514; GMD 5513; GMD 5512; GMD 20047

Fieldwork: La Palma: April 2013, june 2015-11-25. Juana Mª González-Mancebo & Julio Leal; Tenerife: May 2007 Juana Mª González-Mancebo, Jairo Patiño & Gerard Dirkse , March 2011, September 2012. Juana Mª González-Mancebo; Fuerteventura: October 2010. Juana Mª González-Mancebo, Jairo Patiño, Alain Vanderpoorten & Julio Leal

References: Blockeel, T., 2002. Proceedings of the British Bryological Society: British Bryological Society meeting on Tenerife, Canary Islands, february 2001. Bull. British Bryol. Soc. 78: 3; González-Mancebo, J.M., J. Patiño & G.M. Dirkse. 2011. *Andoa berthelotiana* (Mont.) Ochyr. In Garilleti, R. & B. Albertos (coords.) Atlas de los briófitos amenazados de España. Universitat de València. http://www.uv.es/abraesp; González-Mancebo, J.M., Patiño, J., Leal Perez, J., Scholz, S., Fernandez Lopez, A.B., 2009. In: Beltran, E., et al. (Eds.), Amenazas sobre la flora briofitica de la Isla de Fuerteventura. SOS para los últimos supervivientes del extinto bosque de Jandía. Instituto Estudios Canarios, La Laguna, pp. 517–538; Losada Lima, A., J.M. González-Mancebo & E. Beltrán Tejera.1990. Contribution to the bryological knowledge of the reserve of the Biosphere “El Canal y los Tiles” (La Palma, Canary Islands). Courier Forschungsinstitut Senckenberg 159: 195-198.

***Aphanolejeunea madeirensis* (Schiffn.) Grolle**

Madeira

Vouchers: LISU 148953, LISU 161565, LISU 161566, LISU 161567; MADJ1454; MADJ1575; MADJ1575; MADJ3164; MADJ3165; MADJ3167; MADJ3170

***Bryoxiphium madeirense* Á.Löve & D.Löve**

Madeira

Vouchers: LISU 66603, LISU 162233, LISU 162234, LISU 162443, LISU 242545, LISU 242549; MADS409; MADJ8158; MADJ8042; MADJ7665; MADJ6705; MADJ6540; MADJ6099; MADJ5084

***Cheilolejeunea cedercreutzii* (H.Buch & Perss.) Grolle**

Madeira

Vouchers: LISU 248146, LISU 261070

Cololejeunea schaeferi Grolle

Madeira

Vouchers: LISU 161586, LISU 259778, LISU 259785

The Canaries

Vouchers: GMD 5728; GMD 5724; GMD 22519; GMD 22514; GMD 22515; GMD 22540; GMD 6649; GMD 30213; GMD 22522; GMD 22526; GMD 22546; GMD 22541; GMD 22534; GMD 3034; GMD 3006; GMD 3033; GMD 3035; GMD 3228; GMD 3223; GMD 3360; GMD 3236; GMD 3225; GMD 3241; GMD 22494; GMD 11389; GMD 29287; GMD 11633

Field work: La Palma: May 2007, April 2013, May 2014. González Mancebo & Julio Leal; Tenerife: March, April 2012. Juana Mª González-Mancebo; Gomera: January 2008, April & may 2009. Juana Mª González-Mancebo, Jairo Patiño & Julio Leal; Fuerteventura: October 2010. Juana Mª González-Mancebo, Jairo Patiño, Alain Vanderpoorten & Julio Leal

References: Blockeel T. 2002. British Bryological Society Meeting on Tenerife, Canary Islands, February 2001. *Bulletin of the British Bryological Society* 78: 3-11; Dirkse, G.M., A.C. Bouman & A. Losada-Lima, 1993. Bryophytes of the Canary Islands, an annotated check-list. *Cryptogamie, Bryol. Lichénol*. 14 (1): 1- 47; González-Mancebo, J.M. & C.D. Hernández-García 1996. Bryophyte life strategies along an altitudinal gradient in El Canal y los Tiles (La Palma, Canary Islands). *Journal of Bryology* 19: 243-255; González-Mancebo JM, Losada-Lima A, Patiño J, Leal J (2008b) Los briófitos del Parque Nacional de Garajonay. In: Beltrán E (ed) Hongos, líquenes y briófitos del Parque Nacional de Garajonay. Organismo Autónomo de Parques Nacionales, Madrid; González-Mancebo J.M., Albertos B., Barrón A., Cezón K., Cros R.M., Draper I., Estébanez B., Garilleti R., Hallingbäck T., Hernández-Maqueda R., Lara F., Losada Lima A., Mateo R.G., Mazimpaka V., Muñoz J., Medina R., Medina, N.G., Patiño Llorente J., Puche, F., Rams S., Ros R.M., & Ruiz E., 2007. Bryophytes collected by the Spanish Bryological Society during a field trip at La Gomera (Canary Islands). *Boletín de la Sociedad Española de Briología* 30/31: 43-51; González-Mancebo JM, Losada-Lima A, Mcalister S (2003). Host specificity of epiphytic bryophyte communities of a laurel forest on Tenerife (Canary Islands,

Spain). *Bryologist* 106: 383–394; González-Mancebo JM, Romaguera F, Losada-Lima A, Suárez, A (2004) Epiphytic bryophytes growing in *Laurus azorica* (Seub.) Franco in three laurel forest areas in Tenerife (Canary Islands). *Acta Oecologica* 25:159–167; Losada-Lima, A., J.M. González-Mancebo, E. Beltrán Tejera, M.B. Febles-Padilla, M.C. León-Arencibia & A. Bañares Baudet, 1987. Contribución al estudio de los briófitos epífitos del Monte de Aguas y Pasos (Los Silos, Tenerife). I. *Vieraea* 17: 345-352; Losada-Lima, A., J.M. González-Mancebo & E. Beltrán Tejera, 1990. Contribution to the bryological knowledge of the reserve of the Biosphere "El Canal y los Tiles" (La Palma, Canary Islands). *Cour. Forsch.- Inst. Senckenberg* 159: 195-198; Losada-Lima A., González-Mancebo J.M., Febles-Padilla M.B., Beltrán Tejera E., León Arencibia M.C. & Bañares Baudet A., 1990. Contribución al conocimiento de la flora briológica del Monte de Aguas y Pasos (Los Silos, Tenerife). II. Briófitos saxícolas y terrícolas. *Vieraea* 19:11-18; Malme, L., 1988. Distribution of bryophytes on Fuerteventura and Lanzarote, the Canary Islands. *Sommerfeltia* 7: 1-54; Werner J., 2008 - Some remarkable bryophyte records from La Gomera, Tenerife and Madeira. *Cryptogamie Bryologie* 29: 93-98.

***Cryptoleptodon longisetus* (Mont.) Enroth**

Madeira

Vouchers: LISU 149147, LISU 162236, LISU 257607, LISU 259709, LISU 259715, LISU 261252, LISU 261258, LISU 261260

The Canaries

Vouchers: GMD 19896; GMD 19898; GMD 19901;GMD 19886; GMD 19922; GMD 19860; GMD 19926; GMD 19914; GMD 31427; GMD 30223; GMD 19875; GMD 19885; GMD 19865; GMD 19867; GMD 19891; GMD 19883; GMD 29238; GMD 19868; GMD 19881; GMD 29239; GMD 3523; GMD 19858; GMD 3928; GMD 4466; GMD 4468; GMD 19862; GMD 19921; GMD 3665; GMD 19893; GMD 19923; GMD 19869; GMD 19871

Field work: El Hierro: May 2006. Juana Mª González-Mancebo & Julio Leal
La Palma: April 2011, May 2012, June 2013. Juana Mª González-Mancebo, Raquel Hernández Hernández & Julio Leal; Tenerife: March, April 2012. Juana Mª González-Mancebo; Gomera: January 2008, April & may 2009. Juana Mª González-Mancebo, Jairo Patiño & Julio Leal; Gran Canaria: July-Augost 2006, Noviembre 2010, March 2015. Juana Mª González-Mancebo; Fuerteventura: October 2010. Juana Mª González-Mancebo, Jairo Patiño, Alain Vanderpoorten & Julio Leal

References: Ammann, K., L. Sánchez-Pinto & G. Lang,1989. Teneriffa und Gomera 1981-1990; Vegetationsaufnahmen und Artenlisten. 1-56 pp; Blockeel, T., 2002. Proceedings of the British Bryological Society: British Bryological Society meeting on Tenerife, Canary Islands, february 2001. Bull. British Bryol. Soc. 78: 3; Casas, C., 2002. Brioteca Hispánica 1997-1999. Bol. Soc. Esp. Briol. 20/21: 11-21; Dixon, H.N., 1911. Tenerife mosses. Journal of Botany 49: 1-8; Düll, R. 1980. Bryoflora und Bryogeographie der Insel La Palma, Canaren. Cryptogamie, Bryologie-Lichénologie 1: 151-188;nGeheeb, A. & T. Herzog, 1910. Bryologia Atlantica. Die Laubmoose der atlantischen Inseln. Stuttgart; González-Mancebo, J.M. & C.D. Hernández-García 1996. Bryophyte life strategies along an altitudinal gradient in El Canal y los Tiles (La Palma, Canary Islands). Journal of Bryology 19: 243-255; González-Mancebo JM, Losada-Lima A, Mcalister S (2003). Host specificity of epiphytic bryophyte communities of a laurel forest on Tenerife (Canary Islands, Spain). Bryologist 106: 383–394; González-Mancebo JM, Romaguera F, Losada-Lima A, Suárez, A (2004) Epiphytic bryophytes growing in Laurus azorica (Seub.) Franco in three laurel forest areas in Tenerife (Canary Islands). Acta Oecologica 25:159–167; González-Mancebo JM, Losada-Lima A, Patiño J, Leal J (2008b) Los briófitos del Parque Nacional de Garajonay. In: Beltrán E (ed) Hongos, líquenes y briófitos del Parque Nacional de

Garajonay. Organismo Autónomo de Parques Nacionales, Madrid; Gonzalez-Mancebo, J.M., Patiño, J., Leal Perez, J., Scholz, S., Fernandez Lopez, A.B., 2009. In: Beltran, E., et al. (Eds.), Amenazas sobre la flora briofitica de la Isla de Fuerteventura. SOS para los últimos supervivientes del extinto bosque de Jandía; González-Mancebo J.M., Albertos B., Barrón A., Cezón K., Cros R.M., Draper I., Estébanez B., Garilleti R., Hallingbäck T., Hernández-Maqueda R., Lara F., Losada Lima A., Mateo R.G., Mazimpaka V., Muñoz J., Medina R., Medina, N.G., Patiño Llorente J., Puche, F., Rams S., Ros R.M., & Ruiz E., 2007. Bryophytes collected by the Spanish Bryological Society during a field trip at La Gomera (Canary Islands). Boletín de la Sociedad Española de Briología 30/31: 43-51; Losada-Lima & E. Beltrán Tejera, 1987. Estudio de la flora briológica del Monte de Agua García y Cerro del Lomo (Tenerife, Islas Canarias). Anales Jard. Bot. Madrid 44: 233-254; Losada-Lima, A., J.M. González-Mancebo, E. Beltrán Tejera, M.B. Febles-Padilla, M.C. León-Arencibia & A. Bañares Baudet, 1987. Contribución al estudio de los briófitos epífitos del Monte de Aguas y Pasos (Los Silos, Tenerife). I. Vieraea 17: 345-352; Losada-Lima, A., E. Beltrán Tejera, C. Hernández-Padrón & W. Wildpret de la Torre, 1984. Contribución al estudio de los briófitos epífitos de Juniperus phoenicea L. en la isla del Hierro (I. Canarias).1. Anales Biol. Fac. Biol. Univ. Murcia 2 (Sección especial 2): 307-317; Schaminée, J.H.J. & A.H.F. Stortelder 1987. Plantengroei op Tenerife. Verslag van een botanische excursie, april 1986. Rijksinstituut voor onderzoek in de bosen landschapsbouw "De Dorschkamp", Wageningen. Rapport 485; Schwab G., Schäfer-Verwimp, A., Lubenau-Nestle, R. & I. Verwimp, 1986. Beitrag zur Kenntnis der Moosflora der Kanareninsel La Gomera. Bryol. Beitr. 6: 1-31.

Echinodium setigerum **(Mitt.) Jur**

Madeira
Vouchers: MADJ9335; MADJ8432; MADJ8048; MADJ7811; MADJ6363; MADJ6242; MADJ6203; MADJ5958; LISU25681; LISU256721; LISU256720; LISU162111

Echinodium spinosum **(Mitt.) Jur.**

Madeira
Vouchers: LISU 162112, LISU 162113, LISU 162114, LISU 162115, LISU 162116, LISU 162125, LISU 162440, LISU 253310, LISU 253369, LISU 253383, LISU 260775; MADJ5158; MADJ5160; MADJ5161; MADJ5163; MADJ5956; MADJ5957; MADJ5957; MADJ5957; MADJ5959; MADJ6189; MADJ6189; MADJ6202; MADJ6202; MADJ7290; MADJ7400; MADJ7810; MADJ7829; MADJ8034; MADJ8040; MADS1002; MADS1006; MADS4213
The Canaries
Field work: La Palma: April 2010. Juana Mª González-Mancebo & Julio Leal
References: González-Mancebo, J.M., J. Leal 2011; *Echinodium spinosum* (Mitt.) Jur. In Garilleti, R. & B. Albertos (coords.) Atlas de los briófitos amenazados de España. Universitat de València. http://www.uv.es/abraesp

Exsertotheca intermedia (Brid.) S. Olsson, Enroth & D. Quandt

Madeira
Vouchers: LISU 43572, LISU 65546, LISU 149154, LISU 149155, LISU 149156, LISU 149157, LISU 149158, LISU 149159, LISU 162247, LISU 162248, LISU 162250, LISU 162251, LISU 162252, LISU 162253, LISU 162254, LISU 162255, LISU 162256, LISU 162257, LISU 162258, LISU 162259, LISU 162260, LISU 162261, LISU 162262, LISU 174650, LISU 241816, LISU 251167, LISU 251176,

LISU 254370, LISU 254739, LISU 254744, LISU 257588, LISU 257608, LISU 257654, LISU 257692, LISU 257695, LISU 257855, LISU 257939, LISU 257941, LISU 258004, LISU 258136, LISU 258210, LISU 258452, LISU 258453, LISU 258632, LISU 258650, LISU 258825, LISU 258905, LISU 258931, LISU 258947, LISU 258988, LISU 259100, LISU 259182, LISU 259183, LISU 259319, LISU 259329, LISU 259334, LISU 259394, LISU 259418, LISU 259504, LISU 259521, LISU 259551, LISU 259611, LISU 259618, LISU 259632, LISU 259635, LISU 259645, LISU 259737, LISU 259914, LISU 259970, LISU 260003, LISU 260023, LISU 260065, LISU 260099, LISU 260123, LISU 260154, LISU 260211, LISU 260216, LISU 260240, LISU 260262, LISU 260782, LISU 260788, LISU 260842, LISU 260883, LISU 260884, LISU 260908, LISU 261040, LISU 261066, LISU 261077, LISU 261090, LISU 261212

The Canaries

Field work: El Hierro: May 2006. Juana Mª González-Mancebo & Julio Leal
La Palma: April 2011, May 2012, June 2013. Juana Mª González-Mancebo, Raquel Hernández Hernández & Julio Leal; Tenerife: March, April 2012. Juana Mª González-Mancebo; La Gomera: January 2008, April & may 2009. Juana Mª González-Mancebo, Jairo Patiño & Julio Leal; Fuerteventura: October 2010. Juana Mª González-Mancebo, Jairo Patiño, Alain Vanderpoorten & Julio Leal.

References: Ammann, K., L. Sánchez-Pinto & G. Lang,1989. Teneriffa und Gomera 1981-1990, Vegetationsaufnahmen und Artenlisten. 1-56 pp; Blockeel, T., 2002. Proceedings of the British Bryological Society: British Bryological Society meeting on Tenerife, Canary Islands, february 2001. Bull. British Bryol. Soc. 78: 3-11; Boecker, M., E. Fischer & W. Lobin, 1993. Epiphylle Moose von den Kanarischen Inseln (La Gomera und Teneriffa). *Nova Hedwigia* 57 (1-2): 219-230;
Casas, C., 1986. Brioteca Hispanica (1972, 1973, 1974, 1975, 1976-77, 1978-80 y 1984). *Acta Botanica Malacitana* 11: 83-112; Casas, C., 2002. Brioteca Hispánica 1997-1999. *Bol. Soc. Esp. Briol*. 20/21: 11-21; Dixon, H.N., 1911. Tenerife mosses. *Journal of Botany* 49: 1-8; Düll, R. 1980. Bryoflora und Bryogeographie der Insel La Palma, Canaren. Cryptogamie, Bryologie-Lichénologie 1: 151-188;
Geheeb, A. & T. Herzog, 1910. Bryologia Atlantica. Die Laubmoose der atlantischen Inseln. Stuttgart; González-Mancebo, J.M., E. Beltrán Tejera, A.Losada-Lima & L. Sánchez-Pinto, 1996. La vida vegetal en las lavas históricas de Canarias. Colonización y recubrimiento vegetal con especial referencia al Parque Nacional de Timanfaya. Organismo Autónomo de Parques Nacionales; González-Mancebo, J.M. & C.D. Hernández-García 1996. Bryophyte life strategies along an altitudinal gradient in El Canal y los Tiles (La Palma, Canary Islands). *Journal of Bryology* 19: 243-255; González-Mancebo JM, Losada-Lima A, Mcalister S (2003). Host specificity of epiphytic bryophyte communities of a laurel forest on Tenerife (Canary Islands, Spain). *Bryologist* 106: 383–394; González-Mancebo JM, Romaguera F, Losada-Lima A, Suárez, A (2004) Epiphytic bryophytes growing in *Laurus azorica* (Seub.) Franco in three laurel forest areas in Tenerife (Canary Islands). *Acta Oecologica* 25:159–167; González-Mancebo JM, Losada-Lima A, Patiño J, Leal J (2008b) Los briófitos del Parque Nacional de Garajonay. In: Beltrán E (ed) Hongos, líquenes y briófitos del Parque Nacional de Garajonay. Organismo Autónomo de Parques Nacionales, Madrid; Gonzalez-Mancebo, J.M., Patiño, J., Leal Perez, J., Scholz, S., Fernandez Lopez, A.B., 2009. In: Beltran, E., et al. (Eds.), Amenazas sobre la flora briofitica de la Isla de Fuerteventura. SOS para los últimos supervivientes del extinto bosque de Jandía; González-Mancebo J.M., Albertos B., Barrón A., Cezón K., Cros R.M., Draper I., Estébanez B., Garilleti R., Hallingbäck T., Hernández-Maqueda R., Lara F., Losada Lima A., Mateo R.G., Mazimpaka V., Muñoz J., Medina R., Medina, N.G., Patiño Llorente J., Puche, F., Rams S., Ros R.M., & Ruiz E., 2007. Bryophytes collected by the Spanish Bryological Society during a field trip at La Gomera (Canary Islands).

Boletín de la Sociedad Española de Briología 30/31: 43-51; Hallingbäck, T., 1980. Some noteworthy bryophytes from Tenerife. *J. Bryol*. 11: 335-336. Hansen, A. & P. Sunding, 1994. Botanical bibliography of the Canary Islands. *Sommerfeltia* Supplement 5: 1-116; Koppe, F. & R. Düll, 1982. Beiträge zur Bryologie und Bryogeographie von Tenerife. *Bryol. Beitr*. 1: 37-107; Losada-Lima & E. Beltrán Tejera, 1987. Estudio de la flora briológica del Monte de Agua García y Cerro del Lomo (Tenerife, Islas Canarias). *Anales Jard. Bot. Madrid* 44: 233-254; Losada-Lima, A., J.M. González-Mancebo, E. Beltrán Tejera, M.B. Febles-Padilla, M.C. León-Arencibia & A. Bañares Baudet, 1987. Contribución al estudio de los briófitos epífitos del Monte de Aguas y Pasos (Los Silos, Tenerife). I. *Vieraea* 17: 345-352; Losada-Lima, A., E. Beltrán Tejera, C. Hernández-Padrón & W. Wildpret de la Torre, 1984. Contribución al estudio de los briófitos epífitos de *Juniperus phoenicea* L. en la isla del Hierro (I. Canarias).1. *Anales Biol. Fac. Biol. Univ. Murcia* 2 (Sección especial 2): 307-317; Malme, L., 1988. Distribution of bryophytes on Fuerteventura and Lanzarote, the Canary Islands. *Sommerfeltia* 7: 1-54; Schaminée, J.H.J. & A.H.F. Stortelder 1987. Plantengroei op Tenerife. Verslag van een botanische excursie, april 1986. Rijksinstituut voor onderzoek in de bosen landschapsbouw "De Dorschkamp", Wageningen. Rapport 485; Schwab G., Schäfer-Verwimp, A., Lubenau-Nestle, R. & I. Verwimp, 1986. Beitrag zur Kenntnis der Moosflora der Kanareninsel La Gomera. *Bryol. Beitr*. 6: 1-31; Zippel, E., 1998. Die epiphytische Moosvegetation der Kanarischen Inseln. Soziologie, Struktur und Ökologie. Bryophytorum Bibliotheca 52: 1-149. J.Cramer.

***Fissidens coacervatus* Brugg.-Nann**

Madeira

Vouchers: LISU 3636718, LISU 3637231, LISU 3634121, LISU 3635298; MADJ8484; MADJ6439; MADJ5235

The Canaries

Field work: La Palma: June 1999, April 2010. Juana Mª González-Mancebo & Julio Leal; Gomera: November 2008, May 2009. Juana Mª González-Mancebo & Ángel Fernández-López; Tenerife: January 1993 Carmen Dolores Hernández García & Juana María González-Mancebo

References: Blockeel T., 2002 - British Bryological Society Meeting on Tenerife, Canary Islands, February 2001. Bulletin of the British Bryological Society 78: 3-11; Dirkse, G.M., A.C. Bouman & A. Losada-Lima, 1993. Bryophytes of the Canary Islands, an annotated check-list. *Cryptogamie, Bryol. Lichénol*. 14 (1): 1- 47; González-Mancebo JM, Losada-Lima A, Patiño J, Leal J (2008b) Los briófitos del Parque Nacional de Garajonay. In: Beltrán E (ed) Hongos, líquenes y briófitos del Parque Nacional de Garajonay. Organismo Autónomo de Parques Nacionales, Madrid; González-Mancebo, J.M., J. Leal, R. Hernández-Hernández 2011. *Fissidens coacervatus* Brugg.-Nann. In Garilleti, R. & B. Albertos (coords.) Atlas de los briófitos amenazados de España. Universitat de València. http://www.uv.es/abraesp; Hernández-García, C.D., J.M. González-Mancebo & A. Losada-Lima, 1995. Contribución al estudio florístico de cuevas artificiales (galerías) en la isla de Tenerife (Islas Canarias) II. Vieraea 24: 143-152.

***Fissidens nobreganus* Luisier & P.de la Varde**

Madeira

Vouchers: LISU 164018, LISU 149105; MADJ5219; MADJ5974; MADJ5976; MADJ5978; MADJ5980; MADJ6207; MADJ6539; MADJ6730; MADJ8445

***Fissidens sublineaefolius* (P. de la Varde) Brugg.-Nann.**

Madeira

Vouchers: LISU 149112, LISU 162148, LISU 238723, LISU 238844, LISU 238880, LISU 238906, LISU 239019, LISU 239225, LISU 239239, LISU 239247, LISU 239259, LISU 239284, LISU 239303, LISU 239343, LISU 240089, LISU 240103, LISU 240696, LISU 240747, LISU 240790, LISU 240815, LISU 240962, LISU 241022, LISU 241059, LISU 241244, LISU 241274, LISU 241404, LISU 241473, LISU 241496, LISU 241503, LISU 241541, LISU 241567, LISU 241569, LISU 241694, LISU 241758, LISU 241817, LISU 241936, LISU 242327, LISU 242343, LISU 242351, LISU 242379, LISU 243325, LISU 243437, LISU 243447, LISU 243678, LISU 243719,

***Frullania polysticta* Lindenb**

Madeira

Vouchers: LISU 6147, LISU 6223, LISU 6337, LISU 66604, LISU 156119, LISU 156120, LISU 156122, LISU 156124, LISU 157782, LISU 165812, LISU 165813, LISU 165814, LISU 166299, LISU 166301, LISU 166302, LISU 166303, LISU 166304, LISU 166305, LISU 166308, LISU 166309, LISU 173647, LISU 173649, LISU 173662, LISU 249917, LISU 249918, LISU 251746, LISU 251799, LISU 254103, LISU 254184, LISU 254576, LISU 254625, LISU 254636, LISU 254640, LISU 254650, LISU 254710, LISU 257585, LISU 257614, LISU 257622, LISU 257645, LISU 257655, LISU 257680, LISU 257697, LISU 257868, LISU 257980, LISU 258000, LISU 258126, LISU 258758, LISU 258893, LISU 258912, LISU 258930, LISU 258977, LISU 259205, LISU 259273, LISU 259292, LISU 259386, LISU 259413, LISU 259489, LISU 259617, LISU 259630, LISU 259637, LISU 259647, LISU 259732, LISU 259774, LISU 259846, LISU 259849, LISU 259947, LISU 259953, LISU 260016, LISU 260018, LISU 260064, LISU 260066, LISU 260226, LISU 260323, LISU 260329, LISU 261011, LISU 261072, LISU 261069, LISU 261067, LISU 261257, LISU 261180, LISU 261012, LISU 261019, LISU 261236, LISU 261049, LISU 261244, LISU 260934, LISU 261165

The Canaries

Field work: El Hierro: May 2006. Juana Mª González-Mancebo & Julio Leal; La Palma: April 2011, May 2012, June 2013. Juana Mª González-Mancebo, Raquel Hernández Hernández & Julio Leal; Tenerife: March, April 2012. Juana Mª González-Mancebo; Gomera: January 2008, April & may 2009. Juana Mª González-Mancebo, Jairo Patiño & Julio Leal; Gran Canaria: July-Augost 2006, Noviembre 2010, March 2015. Juana Mª González-Mancebo; Fuerteventura: October 2010. Juana Mª González-Mancebo, Jairo Patiño, Alain Vanderpoorten & Julio Leal.

References: Arnell, S., 1961. List of the Hepaticae of the Canary Islands. *Svensk Bot. Tidskr*. 55: 379-393; Blockeel T., 2002 - British Bryological Society Meeting on Tenerife, Canary Islands, February 2001. Bulletin of the British Bryological Society 78: 3-11; Casas C., 1993. Brioteca Hispanica (1987,1988, 1989, 1990). *Bol. Soc. Esp. Briol*. 2: 2-12; Casas, C., M. Brugués, R.M. Cros & C. Sérgio, 1996. Bryophytes Cartography, Iberian peninsula, Balearic and Canary Islands, Azores and Madeira. Fasc.IV: 151-200. Institut d¨Estudis Catalans. Barcelona; Düll, R. 1980. Bryoflora und Bryogeographie der Insel La Palma, Canaren. Cryptogamie, Bryologie-Lichénologie 1: 151-188; González-Mancebo, J.M. & C.D. Hernández-García 1996. Bryophyte life strategies along an altitudinal gradient in El Canal y los Tiles (La Palma, Canary

Islands). *Journal of Bryology* 19: 243-255; González-Mancebo, J.M., E. Beltrán Tejera, A.Losada-Lima & L. Sánchez-Pinto, 1996. La vida vegetal en las lavas históricas de Canarias. Colonización y recubrimiento vegetal con especial referencia al Parque Nacional de Timanfaya. Organismo Autónomo de Parques Nacionales; González-Mancebo JM, Losada-Lima A, Mcalister S (2003). Host specificity of epiphytic bryophyte communities of a laurel forest on Tenerife (Canary Islands, Spain). *Bryologist* 106: 383–394; González-Mancebo JM, Romaguera F, Losada-Lima A, Suárez, A (2004) Epiphytic bryophytes growing in *Laurus azorica* (Seub.) Franco in three laurel forest areas in Tenerife (Canary Islands). *Acta Oecologica* 25:159–167; Gonzalez-Mancebo, J.M., Pati ño, J., Leal Perez, J., Scholz, S., Fernandez Lopez, A.B., 2009a. In: Beltran, E., et al. (Eds.), Amenazas sobre la flora briofitica de la Isla de Fuerteventura. SOS para los últimos supervivientes del extinto bosque de Jandía; González-Mancebo JM, Losada-Lima A, Patiño J, Leal J (2008) Los briófitos del Parque Nacional de Garajonay. In: Beltrán E (ed) Hongos, líquenes y briófitos del;Parque Nacional de Garajonay. Organismo Autónomo de Parques Nacionales, Madrid; González-Mancebo J.M., Albertos B., Barrón A., Cezón K., Cros R.M., Draper I., Estébanez B., Garilleti R., Hallingbäck T., Hernández-Maqueda R., Lara F., Losada Lima A., Mateo R.G., Mazimpaka V., Muñoz J., Medina R., Medina, N.G., Patiño Llorente J., Puche, F., Rams S., Ros R.M., & Ruiz E., 2007. Bryophytes collected by the Spanish Bryological Society during a field trip at La Gomera (Canary Islands). *Boletín de la Sociedad Española de Briología* 30/31: 43-51; Koppe, F. & R. Düll, 1982. Beiträge zur Bryologie und Bryogeographie von Tenerife. *Bryol. Beitr*. 1: 37-107; Losada-Lima & E. Beltrán Tejera, 1987. Estudio de la flora briológica del Monte de Agua García y Cerro del Lomo (Tenerife, Islas Canarias). *Anales Jard. Bot. Madrid* 44: 233-254; Losada-Lima, A., J.M. González-Mancebo, E. Beltrán Tejera, M.B. Febles-Padilla, M.C. León-Arencibia & A. Bañares Baudet, 1987. Contribución al estudio de los briófitos epífitos del Monte de Aguas y Pasos (Los Silos, Tenerife). I. *Vieraea* 17: 345-352; Losada-Lima, A., E. Beltrán Tejera, C. Hernández-Padrón & W. Wildpret de la Torre, 1984. Contribución al estudio de los briófitos epífitos de *Juniperus phoenicea* L. en la isla del Hierro (I. Canarias).1. *Anales Biol. Fac. Biol. Univ. Murcia* 2 (Sección especial 2): 307-317; Malme, L., 1988. Distribution of bryophytes on Fuerteventura and Lanzarote, the Canary Islands. *Sommerfeltia* 7: 1-54; Schaminée, J.H.J. & A.H.F. Stortelder 1987. Plantengroei op Tenerife. Verslag van een botanische excursie, april 1986. Rijksinstituut voor onderzoek in de bosen landschapsbouw "De Dorschkamp", Wageningen. Rapport 485; Schwab, G. & B. Haustein, 1984. Die von dr. W. Hillebrand 1877-1882 auf den Kanaren und Madeiren gesammelten Moose (Bryophyta: Hepaticae et Musci). *Cour. Forsch. Inst. Senckenberg* 71: 113- 124; Schwab G., Schäfer-Verwimp, A., Lubenau-Nestle, R. & I. Verwimp, 1986. Beitrag zur Kenntnis der Moosflora der Kanareninsel La Gomera. *Bryol. Beitr*. 6: 1-31; Van Dort, K.W. & J.A.W. Nieuwkoop, 2003. De bryologische excursie naar Gran Canaria in 1996. *Buxbaumiella* 64: 11-27; Zippel, E., 1998. Die epiphytische Moosvegetation der Kanarischen Inseln. Soziologie, Struktur und Ökologie. Bryophytorum Bibliotheca 52: 1-149. J.Cramer.

***Grimmia curviseta* Bouman**

The Canaries

Field work: Tenerife: May-June 2011, April 2012. Juana Mª González-Mancebo & Alexandra Rodríguez Romero; La Palma: June 2013 Alexandra Rodríguez Romero, Juana Mª González-Mancebo & Julio Leal

References: Bouman, A.C., 1991. Grimmia curviseta spec. nov. (Musci) a new species from Tenerife. J. Bryol. 16: 379-382; González-Mancebo J.M., Losada Lima A. & Patiño Llorente J., 2004. Briófitos. In: Beltrán Tejera E. (ed), Hongos, líquenes y briófitos del Parque Nacional de la Caldera de Taburiente. Naturaleza y Parques

Nacionales. Madrid, Serie Técnica Organismo Autónomo de Parques Nacionales, pp. 352-458; González-Mancebo, J.M., Rodríguez Romero, A. Dirkse G.M. 2011. *Grimmia curviseta Bouman*. In Garilleti, R. & B. Albertos (coords.) Atlas de los briófitos amenazados de España. Universitat de València. http://www.uv.es/abraesp.

***Hedenasiastrum percurrens* (Hedenäs) Ignatov & Vanderpoorten**

Madeira

Vouchers: LISU 162003, LISU 251530, LISU 254715, LISU 257629, LISU 259526, LISU 260846SB8590 SB8588; SB8587; SB8586; SB8585; SB8576; SB8574; SB8571 SB4588; SB4588; SB4535; MADS330; MADJ7375

***Heteroscyphus denticulatus* (Mitt.) Schiffn**

Madeira

Vouchers: LISU 161653, LISU 238847, LISU 254445, LISU 254466, LISU 254474, LISU 254615, LISU 257644, LISU 258003, LISU 258007, LISU 258037, LISU 258041, LISU 258043, LISU 258131, LISU 258179, LISU 258229, LISU 258677, LISU 258911

The Canaries

Vouchers: GMD 7697; GMD 7698; GMD 7699; GMD 7700; GMD 7701; GMD 19481; GMD 11929; GMD 7703; GMD 3086; GMD 3084; GMD 4129; GMD 3317; GMD 3384; GMD 3963; GMD 3696; GMD 5381; GMD 5385; GMD 19479; GMD 19483; GMD 19488; GMD 11391; GMD 28825

Field work: La Palma: April 2011, May 2012, June 2013. Juana Mª González-Mancebo, Raquel Hernández Hernández & Julio Leal; Tenerife: March, April 2012. Juana Mª González-Mancebo; Gomera: January 2008, April & may 2009. Juana Mª González-Mancebo, Jairo Patiño & Julio Leal; Gran Canaria: July-Augost 2006, Noviembre 2010, March 2015. Juana Mª González-Mancebo.

References: Blockeel T., 2002 - British Bryological Society Meeting on Tenerife, Canary Islands, February 2001. Bulletin of the British Bryological Society 78: 3-11; Düll, R. 1980. Bryoflora und Bryogeographie der Insel La Palma, Canaren. Cryptogamie, Bryologie-Lichénologie 1: 151-188; González-Mancebo, J.M. & C.D. Hernández-García 1996. Bryophyte life strategies along an altitudinal gradient in El Canal y los Tiles (La Palma, Canary Islands). *Journal of Bryology* 19: 243-255; González-Mancebo JM, Losada-Lima A, Patiño J, Leal J (2008) Los briófitos del Parque Nacional de Garajonay. In: Beltrán E (ed) Hongos, líquenes y briófitos del Parque Nacional de Garajonay. Organismo Autónomo de Parques Nacionales, Madrid; Hernández-García, C.D., J.M. González-Mancebo & A.Losada-Lima, 1991. A contribution to the study of the flora of artificial caves on the island of Tenerife (Canary Islands). *Mémoires de Biospéologie*, 18: 199-204; Koppe, F. & R. Düll, 1982. Beiträge zur Bryologie und Bryogeographie von Tenerife. *Bryol. Beitr*. 1: 37-107; Losada-Lima, A., J.M. González-Mancebo & E. Beltrán Tejera, 1990. Contribution to the bryological knowledge of the reserve of the Biosphere “El Canal y los Tiles” (La Palma, Canary Islands). *Cour. Forsch.- Inst. Senckenberg* 159: 195-198; Losada-Lima & E. Beltrán Tejera, 1987. Estudio de la flora briológica del Monte de Agua García y Cerro del Lomo (Tenerife, Islas Canarias). *Anales Jard. Bot. Madrid* 44: 233-254; Losada-Lima A., González-Mancebo J.M., Febles-Padilla M.B., Beltrán Tejera E., León Arencibia M.C. & Bañares Baudet A., 1990. Contribución al conocimiento de la flora briológica del Monte de Aguas y Pasos (Los Silos, Tenerife). II. Briófitos saxícolas y terrícolas. *Vieraea* 19:11-18; Schaminée, J.H.J. & A.H.F. Stortelder 1987. Plantengroei op Tenerife. Verslag van een botanische excursie, april 1986. Rijksinstituut voor onderzoek in de bosen landschapsbouw "De Dorschkamp",

Wageningen. Rapport 485; Schwab, G. & B. Haustein, 1984. Die von dr. W. Hillebrand 1877-1882 auf den Kanaren und Madeiren gesammelten Moose (Bryophyta: Hepaticae et Musci). *Cour. Forsch. Inst. Senckenberg* 71: 113- 124; Schwab G., Schäfer-Verwimp, A., Lubenau-Nestle, R. & I. Verwimp, 1986. Beitrag zur Kenntnis der Moosflora der Kanareninsel La Gomera. *Bryol. Beitr*. 6: 1-31; Van Dort, K.W. & J.A.W. Nieuwkoop, 2003. De bryologische excursie naar Gran Canaria in 1996. *Buxbaumiella* 64: 11-27; Zippel, E., 1998. Die epiphytische Moosvegetation der Kanarischen Inseln. Soziologie, Struktur und Ökologie. Bryophytorum Bibliotheca 52: 1-149. J.Cramer.

Homalothecium mandonii

Madeira
Vouchers: LISU 162199, LISU 178973, LISU 162201, LISU 162200, LISU 178896, LISU 173972, LISU 259137, LISU 178895

The Canaries
Field work: El Hierro: May 2006. Juana Mª González-Mancebo & Julio Leal
La Palma: April 2011, May 2012, June 2013. Juana Mª González-Mancebo, Raquel Hernández Hernández & Julio Leal; Tenerife: March, April 2012. Juana Mª González-Mancebo; Gomera: January 2008, April & may 2009. Juana Mª González-Mancebo, Jairo Patiño & Julio Leal; Gran Canaria: July-Augost 2006, Noviembre 2010, March 2015. Juana Mª González-Mancebo; Fuerteventura: October 2010. Juana Mª González-Mancebo, Jairo Patiño, Alain Vanderpoorten & Julio Leal.
References: Hedenas, L., Desamoré, A., Laenen, B., Papp, B. Quandt, D., González-Mancebo, J.M., Patiño, J. Vanderpoorten, A. & M. Stech. 2014. Three species for the price of one within the moss *Homalothecium sericeum* s.l. *Taxon*; 9: 249-257; Malme, L., 1988. Distribution of bryophytes on Fuerteventura and Lanzarote, the Canary Islands. *Sommerfeltia* 7: 1-54.

***Isothecium prolixum* (Mitt.) Stech, Sim-Sim, Tangney & D. Quandt**

Madeira
Vouchers: LISU 149088, LISU 149089, LISU 162437, LISU 162438, LISU 162439, LISU 241501, LISU 241746, LISU 241791, LISU 242201, LISU 242269, LISU 243736, LISU 253211, LISU 253213, LISU 253214, LISU 253215, LISU 253216, LISU 253218, LISU 253219, LISU 253220, LISU 253221, LISU 253222, LISU 253224, LISU 253225, LISU 253317, LISU 253321, LISU 253322, LISU 253328, LISU 253331, LISU 253332, LISU 253373, LISU 253374, LISU 253377, LISU 254776, LISU 257667, LISU 257721, LISU 257941, LISU 257944, LISU 258088, LISU 258106, LISU 258108, LISU 258113, LISU 258436, LISU 258656, LISU 258775, LISU 258780, LISU 258874, LISU 258882, LISU 258971, LISU 259108, LISU 259109, LISU 259135, LISU 259254, LISU 259255, LISU 259265, LISU 259275, LISU 259280, LISU 259387, LISU 259786, LISU 260801

***Isothecium prolixum* (Mitt.) Stech, Sim-Sim, Tangney & D. Quandt**

Madeira
Vouchers: LISU 163298, LISU 259695, LISU 260969

***Leucodon canariensis* (Brid.) Schwägr.**

Madeira
Vouchers: LISU 66603, LISU 162233, LISU 162234, LISU 162443, LISU 242545, LISU 242549, LISU 259439, LISU 260049, LISU 261132; MADJ253; MADJ5419;

MADJ5420; MADJ5422; MADS1816; MADS3772; SB4569; SB9645 SB9646

The Canaries

Vouchers: GMD 19952; GMD 19951; GMD 19957; GMD 19953; GMD 19955; GMD 19949; GMD 19958; GMD 19959; GMD 19961; GMD 19962; GMD 4478; GMD 3981; GMD 3538; GMD 19964; GMD 19967; GMD 16781; GMD 16783; GMD 3932; GMD 3703; GMD 27102; GMD 30626; GMD 30954; GMD 31106; GMD 30164.

Field work: El Hierro: May 2006. Juana Mª González-Mancebo & Julio Leal
La Palma: April 2011, May 2012, June 2013. Juana Mª González-Mancebo, Raquel Hernández Hernández & Julio Leal; Tenerife: March, April 2012. Juana Mª González-Mancebo; Gomera: January 2008, April & may 2009. Juana Mª González-Mancebo, Jairo Patiño & Julio Leal; Gran Canaria: July-Augost 2006, Noviembre 2010, March 2015. Juana Mª González-Mancebo; Fuerteventura: October 2010. Juana Mª González-Mancebo, Jairo Patiño, Alain Vanderpoorten & Julio Leal.

References: Blockeel T., 2002 - British Bryological Society Meeting on Tenerife, Canary Islands, February 2001. Bulletin of the British Bryological Society 78: 3-11; Casas C., 1993. Brioteca Hispanica (1987,1988, 1989, 1990). *Bol. Soc. Esp. Briol*. 2: 2-12; Casas C., 1993. Brioteca Hispanica (1991,1992). *Bol. Soc. Esp. Briol*. 3: 2-7; Düll, R. 1980. Bryoflora und Bryogeographie der Insel La Palma, Canaren. Cryptogamie, Bryologie-Lichénologie 1: 151-188; González-Mancebo, J.M., Patiño, J., Werner, O., Gabriel, R. & Ros, R.M. 2009. Distribution patterns of *Leucodon* species in Macaronesia, with special reference to the Canary Islands. Cryptog. Bryol. 30: 185–197; González-Mancebo, J.M., E. Beltrán Tejera, A.Losada-Lima & L. Sánchez-Pinto, 1996. La vida vegetal en las lavas históricas de Canarias. Colonización y recubrimiento vegetal con especial referencia al Parque Nacional de Timanfaya. Organismo Autónomo de Parques Nacionales; González-Mancebo JM, Losada-Lima A, Mcalister S (2003). Host specificity of epiphytic bryophyte communities of a laurel forest on Tenerife (Canary Islands, Spain). *Bryologist* 106: 383–394; González-Mancebo JM, Romaguera F, Losada-Lima A, Suárez, A (2004) Epiphytic bryophytes growing in *Laurus azorica* (Seub.) Franco in three laurel forest areas in Tenerife (Canary Islands). *Acta Oecologica* 25:159–167; Gonzalez-Mancebo, J.M., Patiño, J., Leal Perez, J., Scholz, S., Fernandez Lopez, A.B., 2009a. In: Beltran, E., et al. (Eds.), Amenazas sobre la flora briofitica de la Isla de Fuerteventura. SOS para los últimos supervivientes del extinto bosque de Jandía. Koppe, F. & R. Düll, 1982. Beiträge zur Bryologie und Bryogeographie von Tenerife. *Bryol. Beitr*. 1: 37-107; Koppe, F. & R. Düll, 1986. Beiträge zur Moosflora von Gran Canaria. *Bryol. Beitr*. 6: 49-57; Long, D.G., 1978. Some noteworthy bryophytes from La Palma, Canary Islands. J. Bryol. 10: 211-212; Schwab, G. & B. Haustein, 1984. Die von dr. W. Hillebrand 1877-1882 auf den Kanaren und Madeiren gesammelten Moose (Bryophyta: Hepaticae et Musci). *Cour. Forsch. Inst. Senckenberg* 71: 113- 124; Schwab G., Schäfer-Verwimp, A., Lubenau-Nestle, R. & I. Verwimp, 1986. Beitrag zur Kenntnis der Moosflora der Kanareninsel La Gomera. *Bryol. Beitr*. 6: 1-31; Zippel, E., 1998. Die epiphytische Moosvegetation der Kanarischen Inseln. Soziologie, Struktur und Ökologie. Bryophytorum Bibliotheca 52: 1-149. J.Cramer.

***Leucodon treleasei* (Cardot)**

Madeira

Vouchers: LISU 251509, LISU 258950, LISU 257582, LISU 257627, LISU 259302, LISU 259214, LISU 259219, LISU 260344, LISU 260026, LISU 260909, LISU 261262, LISU 261208, LISU 260989, LISU 261219, LISU 261259, LISU 261255, LISU 261169, LISU 251509; SB9694; SB9692; SB9690; SB9689; SB9683; SB9676; SB9673; SB9668; SB9665; SB9662; SB4521; SB22458

The Canaries

Vouchers: GMD 8420; GMD 8390; GMD 8393; GMD 16765; GMD 8395; GMD 8380; GMD 16790; GMD 8382; GMD 8396; GMD 4484; GMD 8397; GMD 8391; GMD 19946; GMD 25578; GMD 25529; GMD 27563; GMD 27064; GMD 28720.
Field work: La Palma: April 2011, May 2012, June 2013. Juana Mª González-Mancebo, Raquel Hernández Hernández & Julio Leal; Tenerife: April 2009. Juana Mª González-Mancebo & Jairo Patiño.
References: Bryhn, N., 1908. Ad cognitionem bryophytorum archipelagi canariensis contributio. *Kongel. Norske Vidensk. Selsk. Skr*. 8: 1-35; González-Mancebo, J.M., Patiño, J., Werner, O., Gabriel, R. & Ros, R.M. 2009. Distribution patterns of Leucodon species in Macaronesia, with special reference to the Canary Islands. Cryptog. Bryol. 30: 185–197.

Pelekium atlanticum (Hedenäs) Hedenäs

Madeira
Vouchers: LISU 162364, LISU 162365, LISU 162366, LISU 162367, LISU 239215, LISU 240711, LISU 240759, LISU 241079, LISU 241229, LISU 241432, LISU 242354, LISU 242392, LISU 243557, LISU 244172, LISU 244195, LISU 244247, LISU 244272, LISU 244336, LISU 245039; MADJ 5883; MADJ2479; MADJ5637; MADJ5640; MADJ5641; MADJ6590; MADJ7061; MADS2467; SB10020; SB10022; SB10025; SB10036; SB10038; SB12093; SB4592; SB8246
The Canaries
Vouchers: GMD 6221; GMD 15909; GMD 6452; GMD 6219; GMD 6458; GMD 6225.
Field work: La Palma: April 2011, May 2012, June 2013. Juana Mª González-Mancebo, Raquel Hernández Hernández & Julio Leal; Tenerife: March, April 2012. Juana Mª González-Mancebo; Gomera: January 2008, April & may 2009. Juana Mª González-Mancebo, Jairo Patiño & Julio Leal
References: Casas C., 1993. Brioteca Hispanica (1987,1988, 1989, 1990). *Bol. Soc. Esp. Briol*. 2: 2-12; Dirkse, G.M., A.C. Bouman & A. Losada-Lima, 1993. Bryophytes of the Canary Islands, an annotated check-list. *Cryptogamie, Bryol. Lichénol*. 14 (1): 1-47; Düll, R. 1980. Bryoflora und Bryogeographie der Insel La Palma, Canaren. Cryptogamie, Bryologie-Lichénologie 1: 151-188; Hallingbäck, T., 1980. Some noteworthy bryophytes from Tenerife. *J. Bryol*. 11: 335-336. Hansen, A. & P. Sunding, 1994. Botanical bibliography of the Canary Islands. *Sommerfeltia* Supplement 5: 1-116; Losada-Lima, A., J.M. González-Mancebo & E. Beltrán Tejera, 1990. Contribution to the bryological knowledge of the reserve of the Biosphere "El Canal y los Tiles" (La Palma, Canary Islands). *Cour. Forsch.- Inst. Senckenberg* 159: 195-198; Losada-Lima A., González-Mancebo J.M., Febles-Padilla M.B., Beltrán Tejera E., León Arencibia M.C. & Bañares Baudet A., 1990. Contribución al conocimiento de la flora briológica del Monte de Aguas y Pasos (Los Silos, Tenerife). II. Briófitos saxícolas y terrícolas. *Vieraea* 19:11-18.

***Plagiochila maderensis* Gottsche ex Steph.**

Madeira
Vouchers: LISU 66707, LISU 66709,
LISU 161838, LISU 161841, LISU 161844, LISU 190980, LISU 190985, LISU 240737, LISU 241226, LISU 241291, LISU 242255, LISU 242284, LISU 242300, LISU 242323, LISU 242360, LISU 243661, LISU 244175, LISU 244236, LISU 244263, LISU 249966, LISU 250009, LISU 250010, LISU 250011, LISU 250012, LISU 250013, LISU 250014, LISU 250015, LISU 250016, LISU 250017, LISU

251719, LISU 251728, LISU 251806, LISU 254091, LISU 254132, LISU 254147, LISU 254199, LISU 254541, LISU 254561, LISU 254599, LISU 254609, LISU 254672, LISU 254697, LISU 254702

The Canaries

Field work: Tenerife: March, April 2012. Juana Mª González-Mancebo; Gomera: January 2008, April & may 2009. Juana Mª González-Mancebo, Jairo Patiño & Julio Leal

References: González-Mancebo JM, Losada-Lima A, Patiño J, Leal J (2008) Los briófitos del Parque Nacional de Garajonay. In: Beltrán E (ed) Hongos, líquenes y briófitos del Parque Nacional de Garajonay. Organismo Autónomo de Parques Nacionales, Madrid; González-Mancebo, J.M., Draper, I., Lara, F., Marrero, J.D., Muñoz, J., Patiño, J., Romaguera, F. & A. Vanderpoorten, 2009. Amendments to the bryophyte flora of the Cape Verde and Canary Islands. *Cryptogamie Bryologie*; González-Mancebo, J.M., Hernández-Hernández, R. & J. Patiño. 2011. *Plagiochila maderensis* Gottsche ex Steph. In Garilleti, R. & B. Albertos (coords.) Atlas de los briófitos amenazados de España. Universitat de València. http://www.uv.es/abraesp.

***Porella inaequalis* (Gottsche ex Steph.) Perss.**

Madeira

Vouchers: LISU 9457, LISU 149003, LISU 163998, LISU 261009; MADJ2652; MADJ2649; MADJ2172; MADJ1168

Radula wichurae Steph.

Madeira

Vouchers: LISU 66725, LISU 161891, LISU 163281, LISU 163283, LISU 183533, LISU 250058, LISU 257632, LISU 259369, LISU 259788, LISU 232875, LISU 232874, LISU 232873, LISU 250058; MADJ3234; MADS227; MADS227; MADS403

The Canaries

Vouchers: GMD 3951

Field work: Tenerife: May 2013. Juana María González Mancebo, Alexandra Rodríguez Romero & Gerard Dirkse;

References: Bouman, A.C. & G.M. Dirkse, 1992. The genus *Radula* in Macaronesia. *Lindbergia* 16: 119-127; González-Mancebo, J.M., Hernández-Hernández, R. & J. Patiño. 2011. *Radula wichurae* Steph. In Garilleti, R. & B. Albertos (coords.) Atlas de los briófitos amenazados de España. Universitat de València. http://www.uv.es/abraesp; Hallingbäck, T., 1980. Some noteworthy bryophytes from Tenerife. *J. Bryol*. 11: 335-336. Hansen, A. & P. Sunding, 1994. Botanical bibliography of the Canary Islands. *Sommerfeltia* Supplement 5: 1-116.

***Rhynchostegiella bourgaeana* (Mitt.) Broth.**

The Canaries

Vouchers: GMD 7909; GMD 7851; GMD 7870; GMD 30599; GMD 7928; GMD 27881; GMD 13831.

Field work: Hierro: March 2010. Juana María González Mancebo, Jairo Patiño & Alain Vanderpoorten; Tenerife: April 2010; November 2011. Jairo Patiño &Juana María González-Mancebo; Gran Canaria: March 2010, 20111. J.M. González-Mancebo

References: Dirkse, G.M. & A.C. Bouman 1995. A revision of *Rhynchostegiella* (Musci, Brachytheciaceae) in the Canary Islands. *Lindbergia* 20: 109-121; Gerard M. Dirkse, Jairo Patiño &Juana María González-Mancebo. 2011. *Rhynchostegiella*

bourgaeana (Mitt.) Broth. In Garilleti, R. & B. Albertos (coords.) Atlas de los briófitos amenazados de España. Universitat de València. http://www.uv.es/abraesp.

***Rhynchostegiella macilenta* (Renauld & Cardot) Cardot**

Madeira

Vouchers: LISU 162338, LISU 162339, LISU 239235, LISU 239292, LISU 242417, LISU 243554, LISU 243957, LISU 243971, LISU 244018, LISU 257603; SB9114; SB9113; SB9111; SB9110; SB9109; SB9102; SB9101; SB9100; SB9099; SB9098; SB9097; SB9096; SB9094

The Canaries

Field work: La Palma: Enero 2012. Juana María González-Mancebo, Jairo Patiño & Julio Leal; Gomera: October-November 2010, December 2011, January 2012. Juana María González-Mancebo, Jairo Patiño & Julio Leal; Tenerife: July, August, 2011; January, February 2012. Juana María González-Mancebo, & Julio Leal.

References:

Blockeel, T., 2002. Proceedings of the British Bryological Society: British Bryological Society meeting on Tenerife, Canary Islands, february 2001. Bull. British Bryol. Soc. 78: 3; Bryhn, N., 1908. Ad cognitionem bryophytorum archipelagi canariensis contributio. Kongel. Norske Vidensk. Selsk. Skr. 8: 1-35; Dirkse, G.M. & A.C. Bouman 1995. A revision of *Rhynchostegiella* (Musci, Brachytheciaceae) in the Canary Islands. *Lindbergia* 20: 109-121; Geheeb, A. & T. Herzog, 1910. Bryologia Atlantica. Die Laubmoose der atlantischen Inseln. Stuttgart. Rhynchostegiella trichophylla Dirkse & Bouman

***Rhynchostegiella trichophylla* Dirkse & Bouman**

Madeira

Vouchers: LISU 239178

The Canaries

Vouchers: GMD 7899; GMD 7900; GMD 13845; GMD 7902; GMD 8267; GMD 7788; GMD 7903; GMD 7789; GMD 3967; GMD 4141; GMD 3974; GMD 7905; GMD 7906; GMD 7824; GMD 8304; GMD 7944; GMD 13864; GMD 13866; GMD 13867; GMD 7943; GMD 13841; GMD 13842; GMD 28882; GMD 26488; GMD 26710; GMD 13868.

References: Dirkse, G.M. & A.C. Bouman 1995. A revision of *Rhynchostegiella* (Musci, Brachytheciaceae) in the Canary Islands. *Lindbergia* 20: 109-121; González-Mancebo, J.M., E. Beltrán Tejera & A.Losada-Lima, 1991. Contribución al estudio de la flora y vegetación briofítica higro-hidrófila de las Cañadas del Teide (Tenerife). Instituto de Estudios Canarios. La Laguna de Tenerife; González-Mancebo JM, Losada-Lima A, Patiño J, Leal J (2008) Los briófitos del Parque Nacional de Garajonay. In: Beltrán E (ed) Hongos, líquenes y briófitos del Parque Nacional de Garajonay. Organismo Autónomo de Parques Nacionales, Madrid

***Telaranea azorica* (H. Buch & Perss.) Schumacker & Váňa**

The Canaries

Field work: Tenerife: Abril, 2011. G.M. Dirkse & J.M. González Mancebo

References: Dirkse G.M, Bouman A.C. & Losada-Lima A., 1993 - Bryophytes of the Canary Islands, an annotated checklist. *Cryptogamie, Bryologie et Lichénologie 14 (1): 1-7;* González-Mancebo, J.M. & Dirkse G.M. 2011. *Telaranea azorica* (H. Buch & Perss.) Pócs ex Schumacker & Váňa. In Garilleti, R. & B. Albertos (coords.) Atlas

	de los briófitos amenazados de España. Universitat de València. http://www.uv.es/abraesp.
***Tortella limbata* (Schiffn.) Geh. & Herzog**	**Madeira**<br>Vouchers: MADS2537; MADS2536; MADS2535; MADS3178; MADS3176; MADS3175; MADS3181; MADS3179; MADS3174<br>**The Canaries**<br>Vouchers: GMD 6391; GMD 28050.<br>References: Cezón, K. & Muñoz, J. 2006. The rediscovery of *Tortella limbata* (Pottiaceae). The Bryologists 109 (3) 401-403; Geheeb, A. & T. Herzog, 1910. Bryologia Atlantica. Die Laubmoose der atlantischen Inseln. Stuttgart; Schiffner, V., 1902. Neue Materialien zur Kenntnis der Bryophyten der atlantischen Inseln. *Hedwigia* 41: 269-294.

Table S2. Number of 1–km^2 climatically suitable pixels for each of 35 investigated Macaronesian endemic bryophyte species in Azores; the Canary Islands and Madeira under present climate conditions (P); and number (percentage of climatically suitable areas as compared to the present situation in both island and continental areas) in 2070 under the climate conditions defined by the RCP 4.5 and RCP 8.5 concentration pathways.

	Azores			Canaries			Madeira		
	P	RCP4.5	RCP8.5	P	RCP4.5	RCP8.5	P	RCP4.5	RCP8.5
Alophosia azorica	2698	1473	451	0	0	0	0	0	0
Andoa berthelotiana	3706	2077	879	0	0	0	0	0	0
Bazzania azorica	2066	572	522	0	0	0	0	0	0
Breutelia azorica	2093	856	428	0	0	0	0	0	0
Bryoxyphium madeirense	2	0	0	147	15	0	1008	334	0
Calypogeia azorica	2353	943	469	0	0	0	0	0	0
Cheilolejeunea cedercreutzii	694	198	161	0	0	0	0	0	0
Cololejeunea schaeferi	0	0	0	1545	350	2	861	322	12
Cryptoleptodon longisetus	0	0	0	2589	828	77	806	637	259
Echinodium renauldii	2783	1046	651	0	0	0	0	0	0
Echinodium setigerum	0	1	0	55	28	0	694	275	0
Echinodium spinosum	0	0	0	90	3	0	944	300	0
Exsertotheca intermedia	1043	2196	740	6019	1403	0	1131	683	0
Fissidens coacervatus	0	0	0	2956	1400	97	1035	802	561
Fissidens nobreganus	2	0	0	107	0	0	980	474	1
Fissidens sublineaefolius	0	0	0	310	0	0	1034	512	0

Table S2. (continued)

	Azores			Canaries			Madeira		
	P	RCP4.5	RCP8.5	P	RCP4.5	RCP8.5	P	RCP4.5	RCP8.5
Frullania polysticta	0	0	0	3945	1409	304	1128	874	586
Grimmia curviseta	0	0	0	679	61	9	0	0	0
Hedenasiastrum percurrens	0	0	0	17	0	0	689	235	8
Heteroscyphus denticulatus	3037	1	601	2616	396	16	1029	653	353
Homalothecium mandonii	0	0	0	5381	3446	2051	907	745	475
Isothecium prolixum	2337	842	454	0	0	0	0	0	0
Leptoscyphus azoricus	802	171	122	0	0	0	0	0	0
Leucodon canariensis	989	0	0	2724	669	82	1123	645	369
Leucodon treleasei	1768	2644	589	0	0	0	0	0	241
Pelekium atlanticum	0	0	0	2253	650	0	1124	710	248
Plagiochila maderensis	0	0	0	1987	770	0	1092	685	252
Porella inaequalis	0	0	0	237	59	12	1131	891	721
Radula wichurae	3506	2239	688	0	0	0	0	0	0
Rhynchostegiella bourgaeana	0	0	0	2446	241	21	122	0	0
Rhynchostegiella macilenta	0	0	0	2373	962	257	1062	761	500
Rhynchostegiella trichophylla	0	0	0	2975	905	201	470	0	0
Riccia atlantica	0	0	0	0	0	0	174	2	0
Telaranea azorica	796	309	162	0	0	0	0	0	0
Tortella limbata	6	0	0	2429	0	54	562	282	222

Figure S1. Detail of the geographic background (i.e. the extent of the study area) defined to calibrate the models of the present study. Each dot represents 10,000 points randomly sampled to decrease the number of correlated bioclimatic variables used for the species distribution models. Maps were created using ArcGIS software by Esri (Environmental Systems Resource Institute; ArcGIS 10.0; www.esri.com).

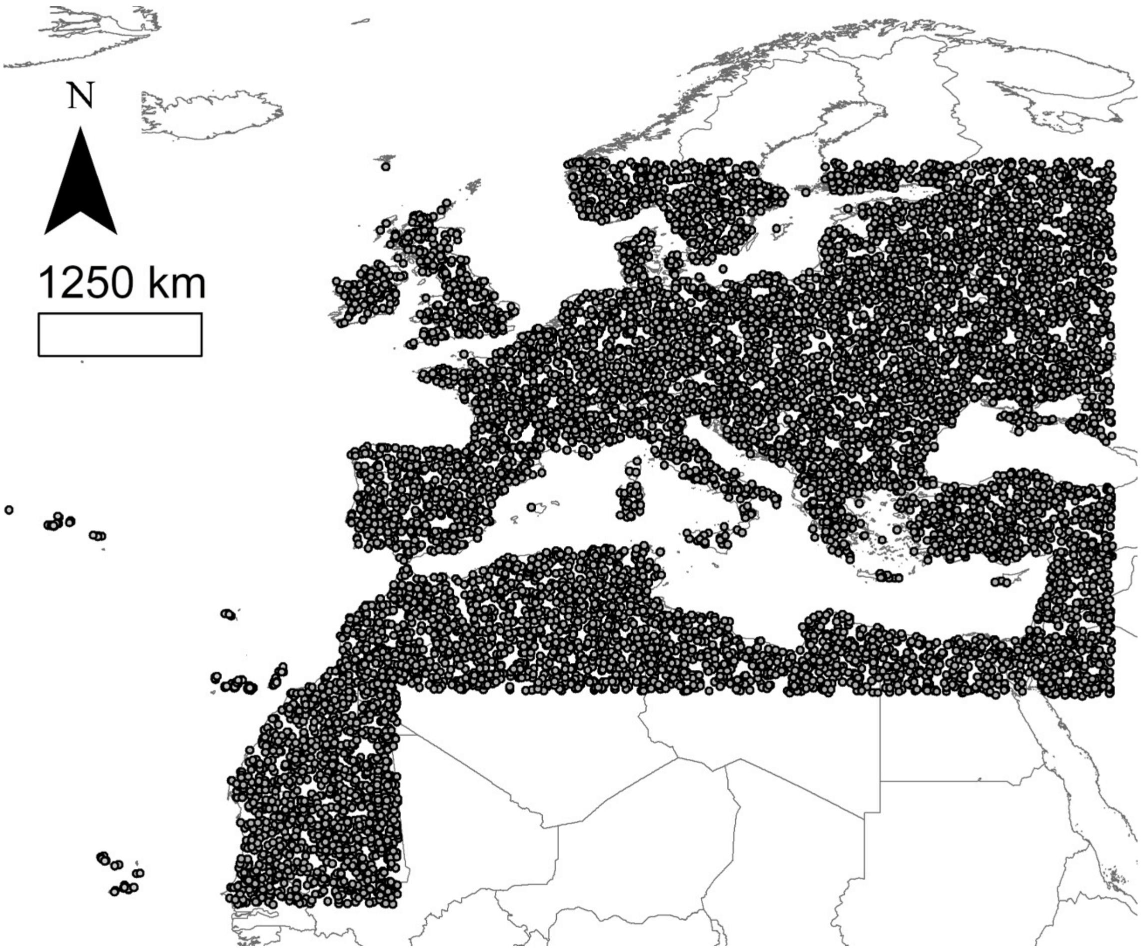

Figure S2. Potential distribution of each of 35 Macaronesian endemic bryophyte species in Macaronesia; western Europe and northwestern Africa under present and future (2070) climate conditions defined by the RCP 4.5 (B) and RCP 8.5 (C) concentration pathways. Crosses indicate extinctions in 2070. Maps were created using ArcGIS software by Esri (Environmental Systems Resource Institute; ArcGIS 10.0; www.esri.com).

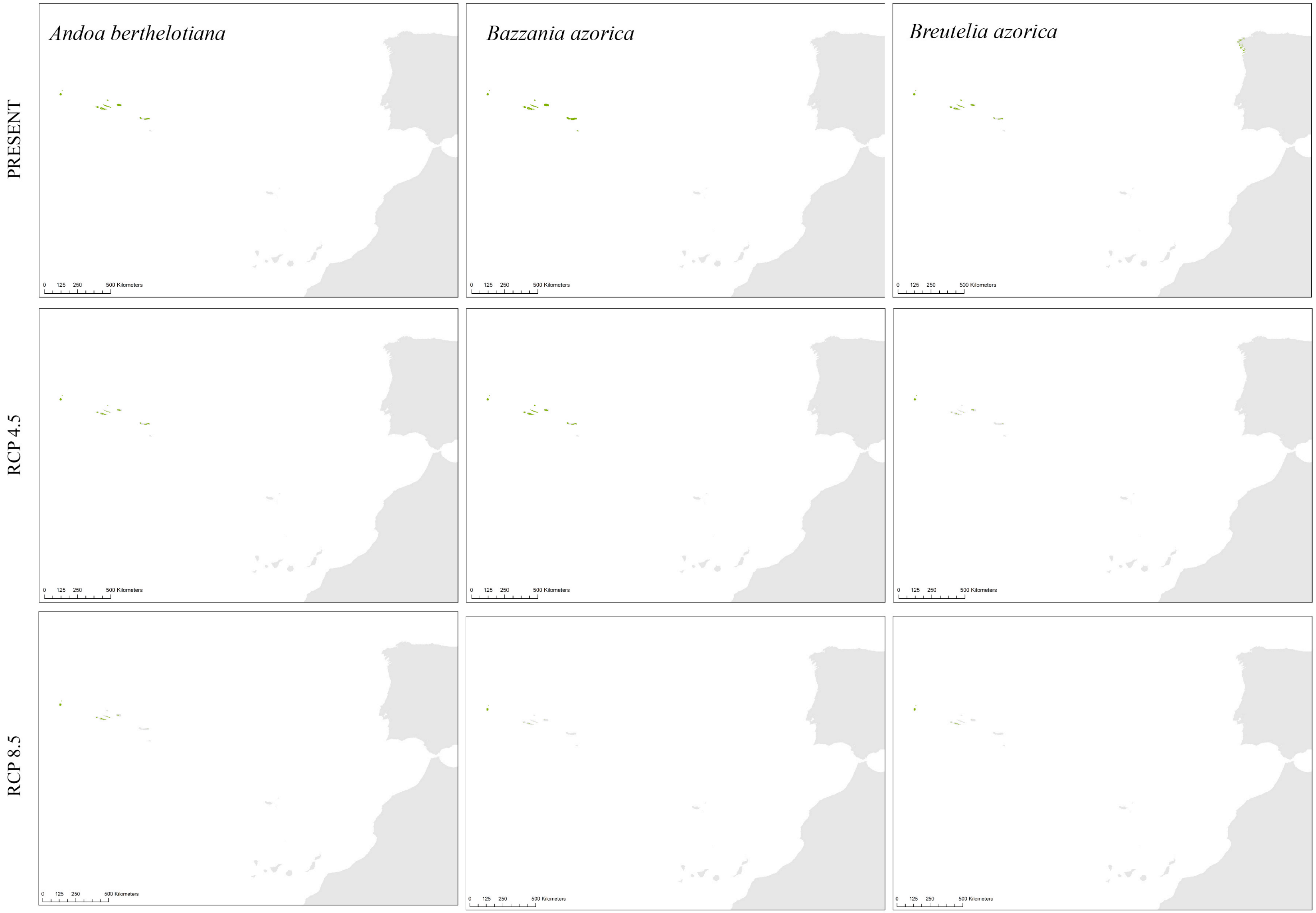

Figure S2 (continued). Maps were created using ArcGIS software by Esri (Environmental Systems Resource Institute; ArcGIS 10.0; www.esri.com).

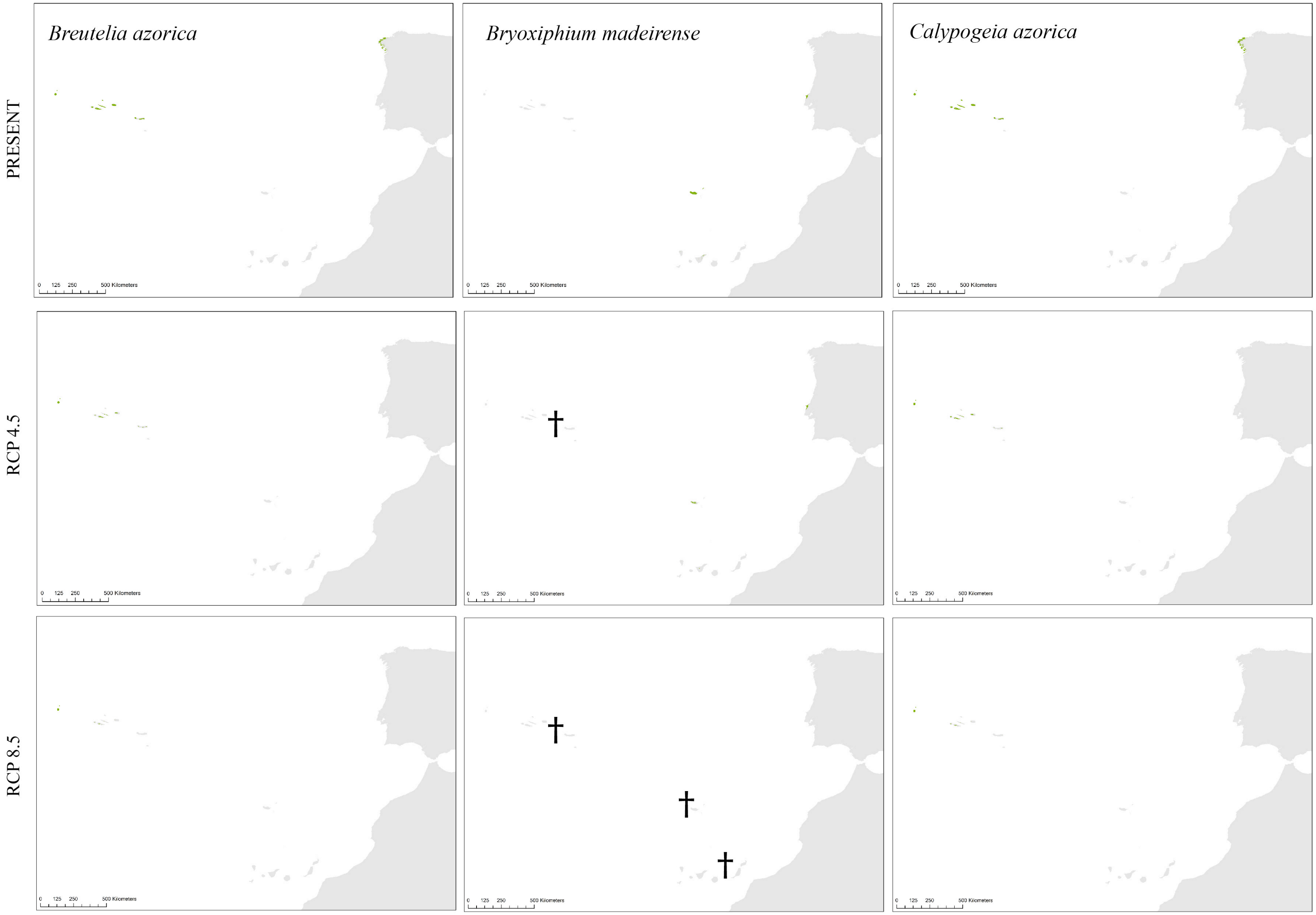

Figure S2 (continued). Maps were created using ArcGIS software by Esri (Environmental Systems Resource Institute; ArcGIS 10.0; www.esri.com).

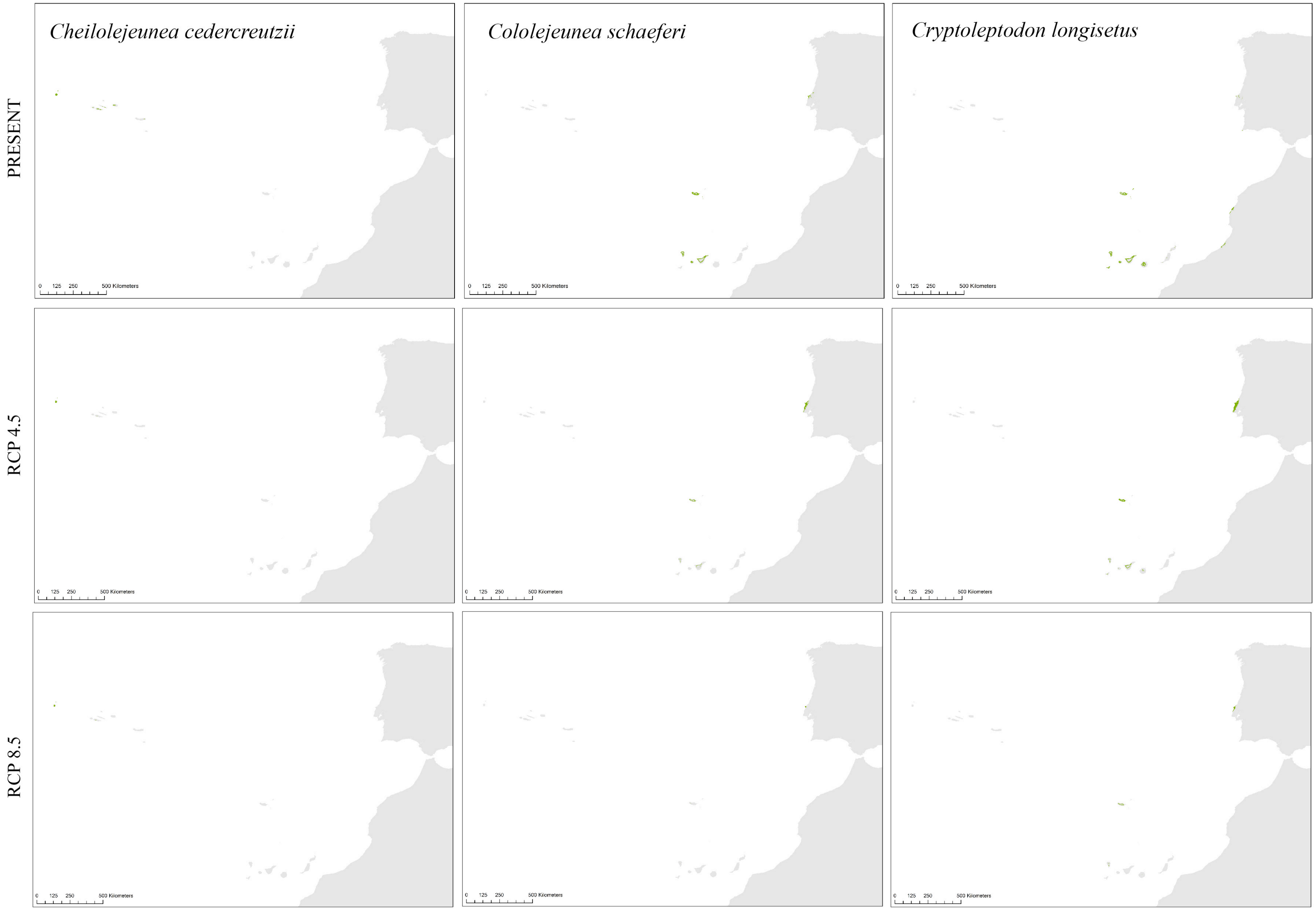

Figure S2 (continued). Maps were created using ArcGIS software by Esri (Environmental Systems Resource Institute; ArcGIS 10.0; www.esri.com).

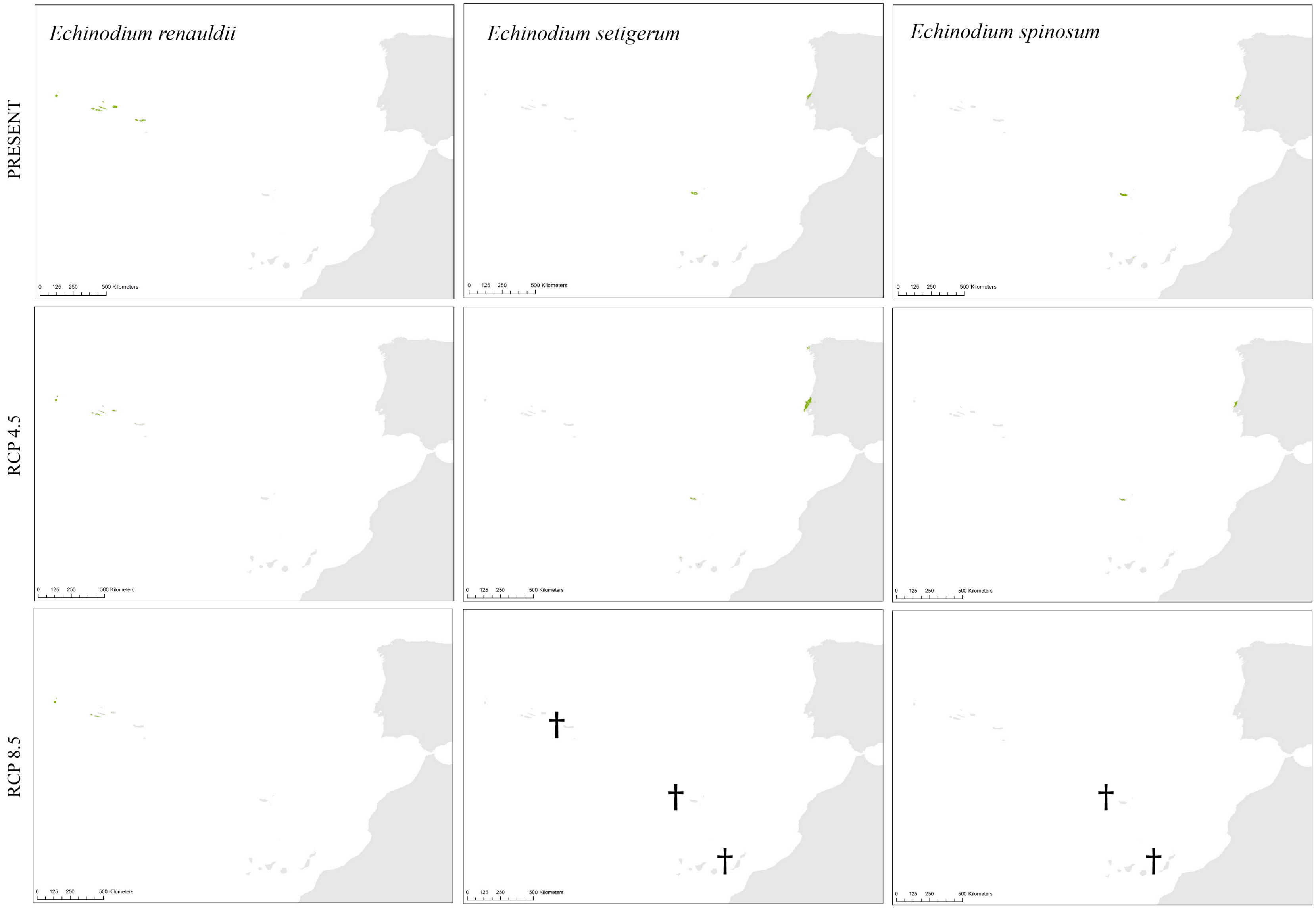

Figure S2 (continued). Maps were created using ArcGIS software by Esri (Environmental Systems Resource Institute; ArcGIS 10.0; www.esri.com).

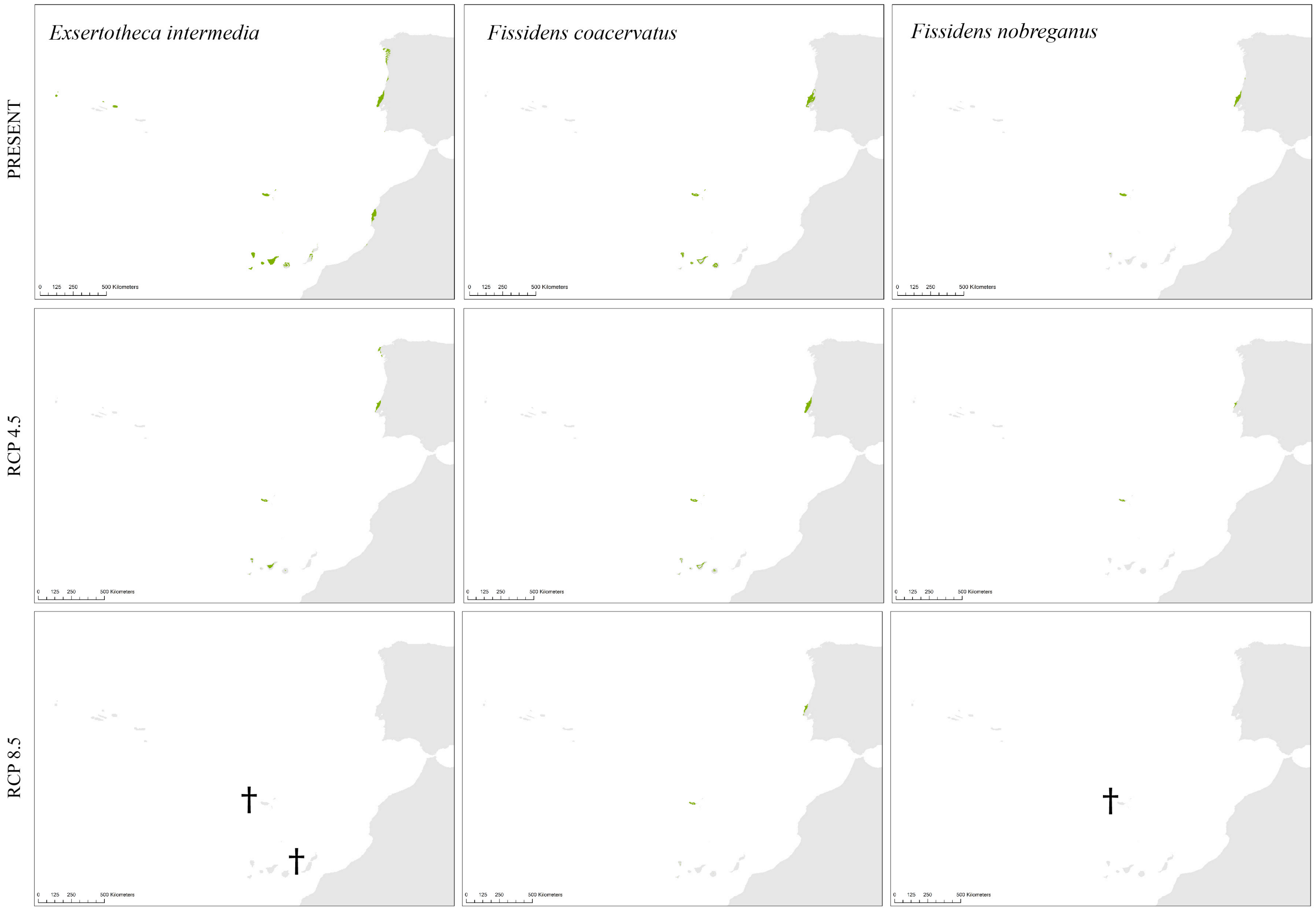

Figure S2 (continued). Maps were created using ArcGIS software by Esri (Environmental Systems Resource Institute; ArcGIS 10.0; www.esri.com).

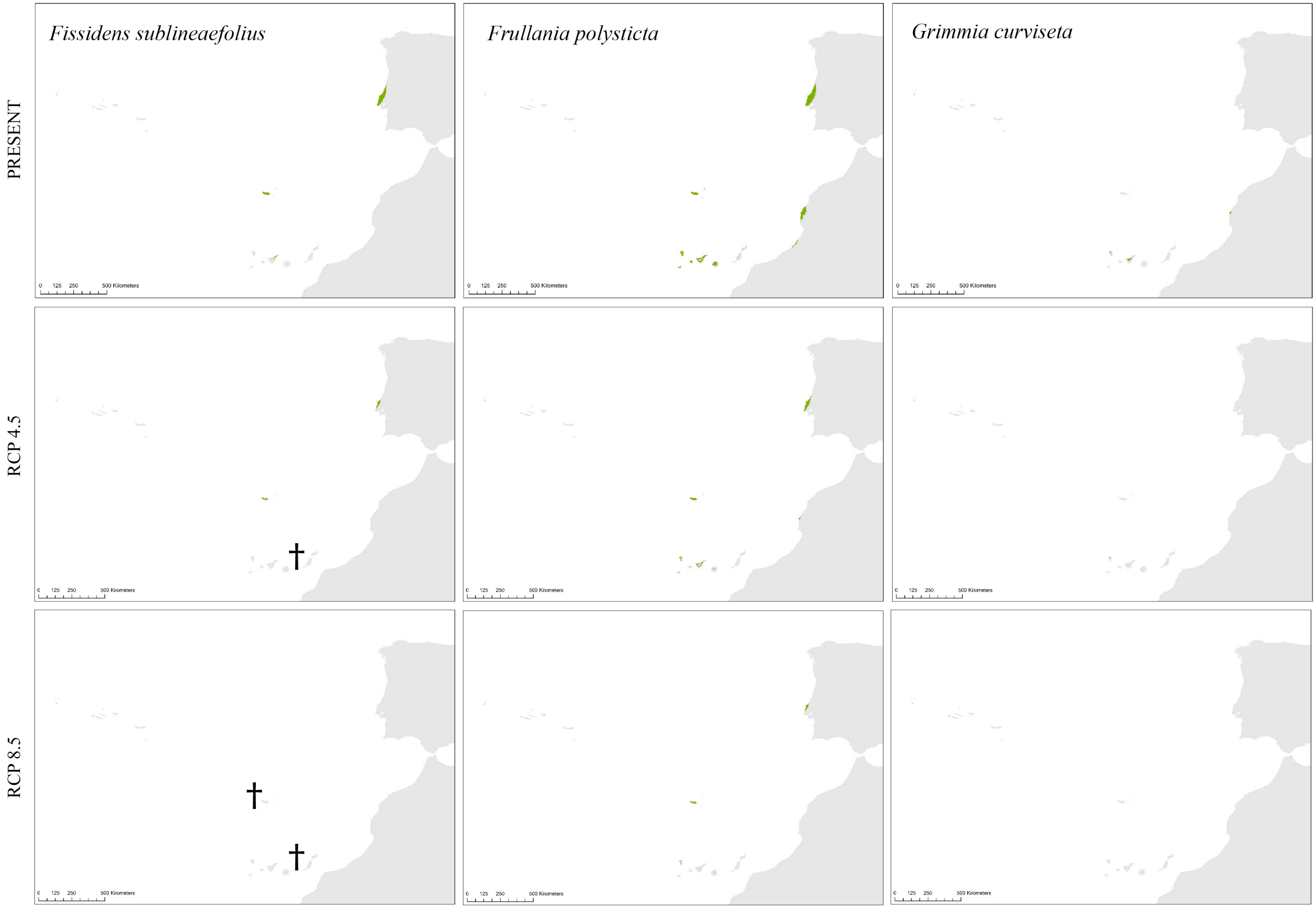

Figure S2 (continued). Maps were created using ArcGIS software by Esri (Environmental Systems Resource Institute; ArcGIS 10.0; www.esri.com).

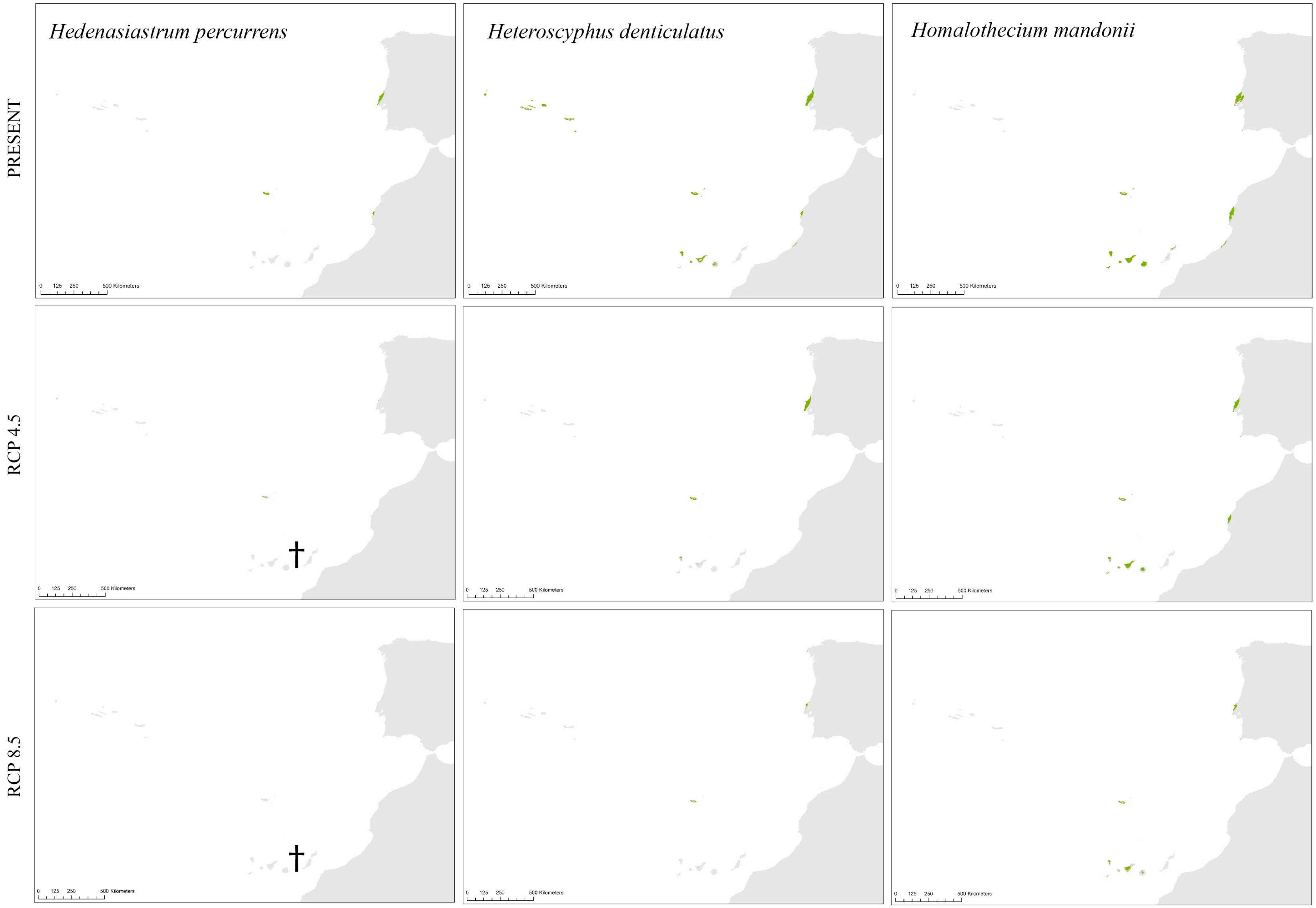

Figure S2 (continued). Maps were created using ArcGIS software by Esri (Environmental Systems Resource Institute; ArcGIS 10.0; www.esri.com).

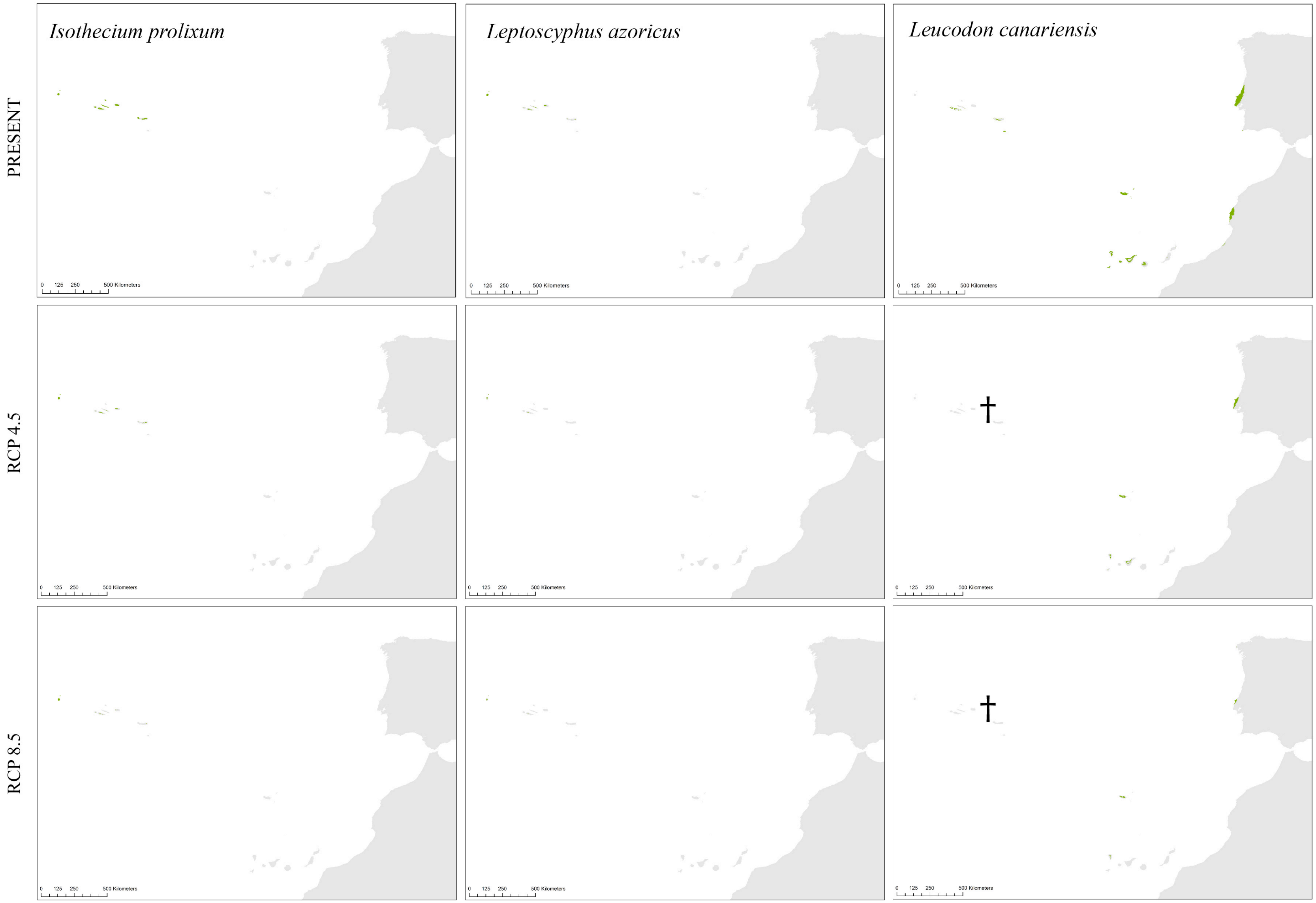

Figure S2 (continued). Maps were created using ArcGIS software by Esri (Environmental Systems Resource Institute; ArcGIS 10.0; www.esri.com).

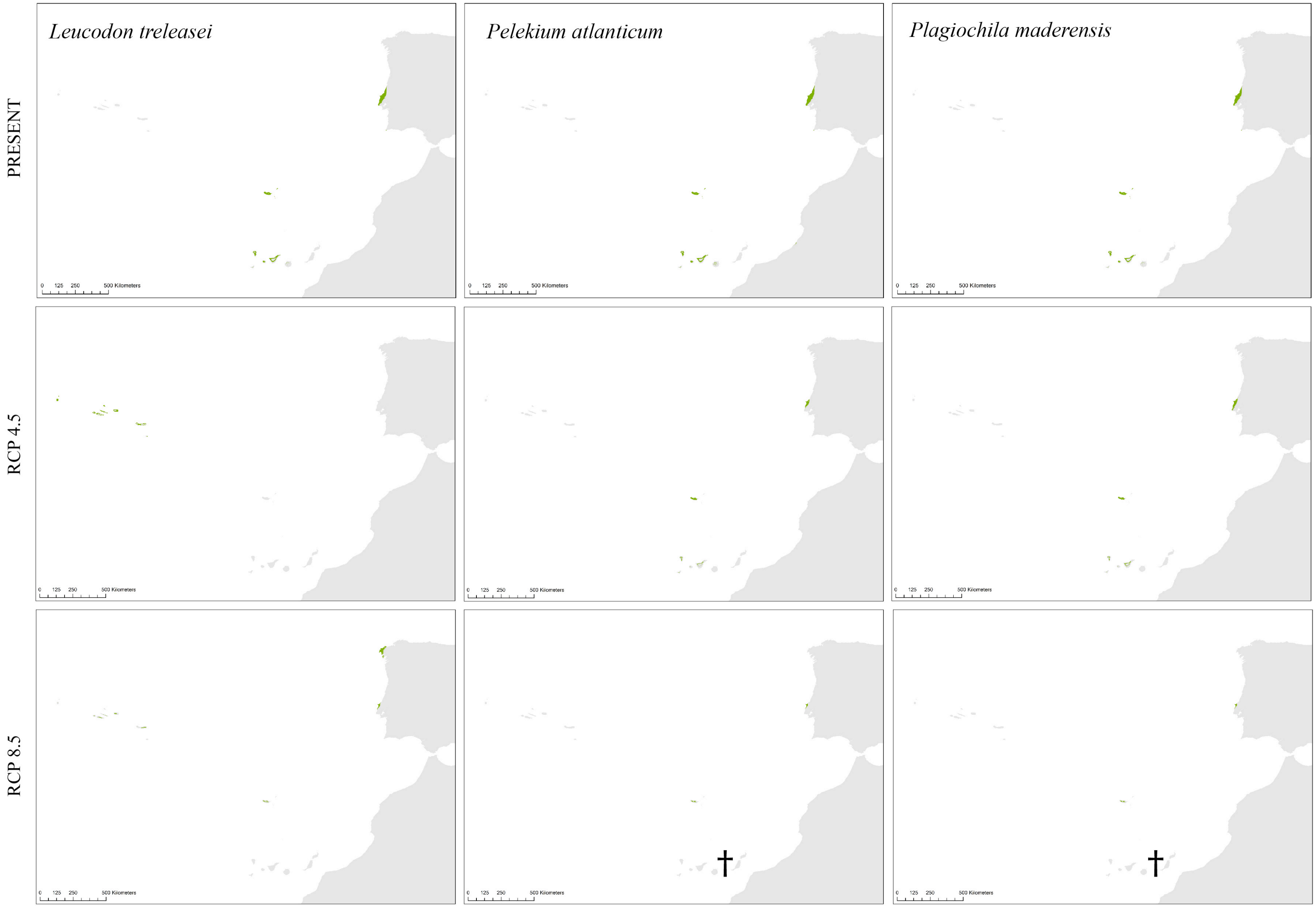

Figure S2 (continued). Maps were created using ArcGIS software by Esri (Environmental Systems Resource Institute; ArcGIS 10.0; www.esri.com).

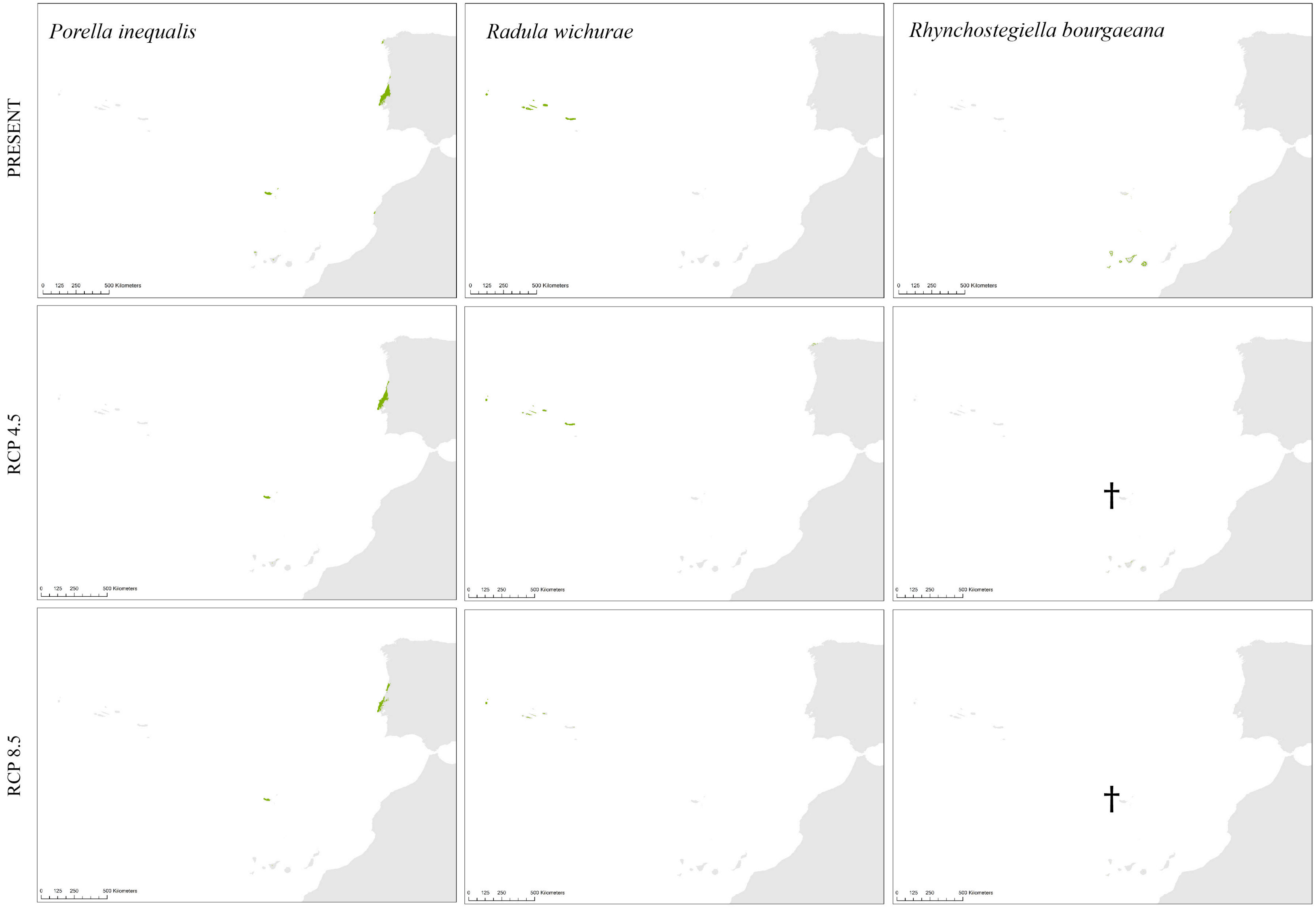

Figure S2 (continued). Maps were created using ArcGIS software by Esri (Environmental Systems Resource Institute; ArcGIS 10.0; www.esri.com).

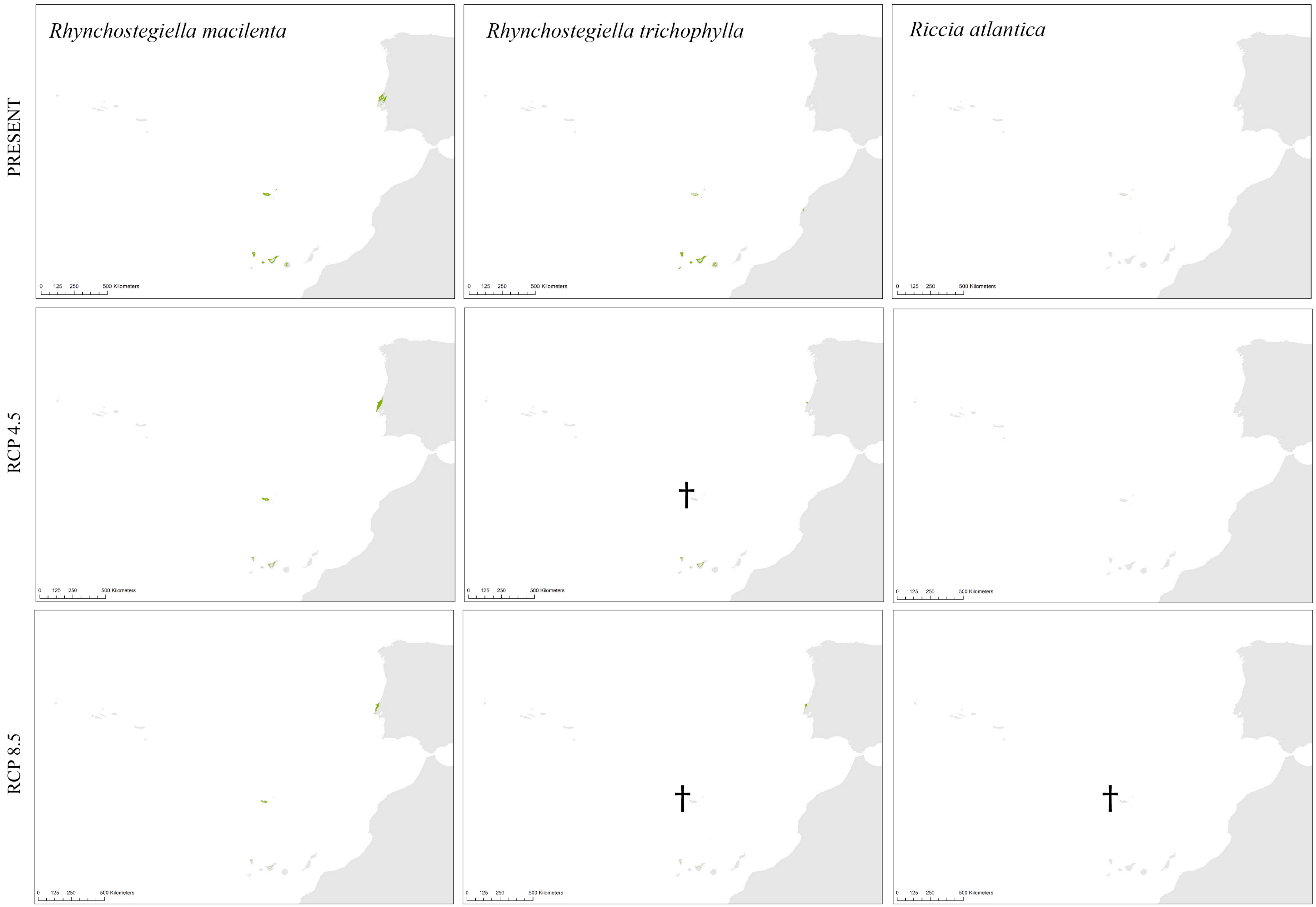

Figure S2 (continued). Maps were created using ArcGIS software by Esri (Environmental Systems Resource Institute; ArcGIS 10.0; www.esri.com).

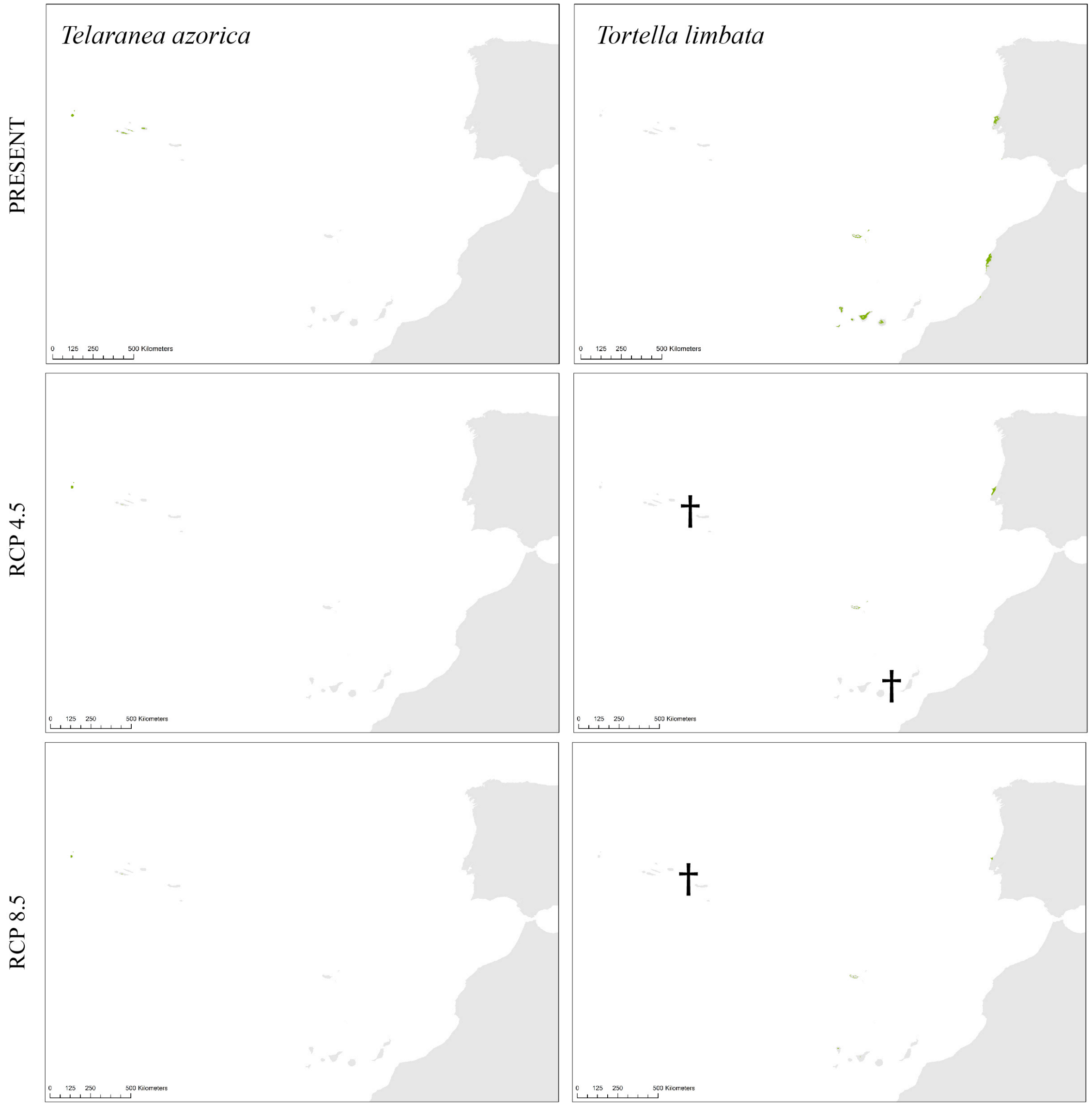

Figure S3. Detail of the identified the continental areas with analogous climates to those of the Macaronesian islands under both present and future conditions using Multivariate Environmental Similarity Surfaces (MESS). Colored pixels indicate areas with analogous climate across Macaronesia and western Europe and North Africa. Maps were created using ArcGIS software by Esri (Environmental Systems Resource Institute; ArcGIS 10.0; www.esri.com).

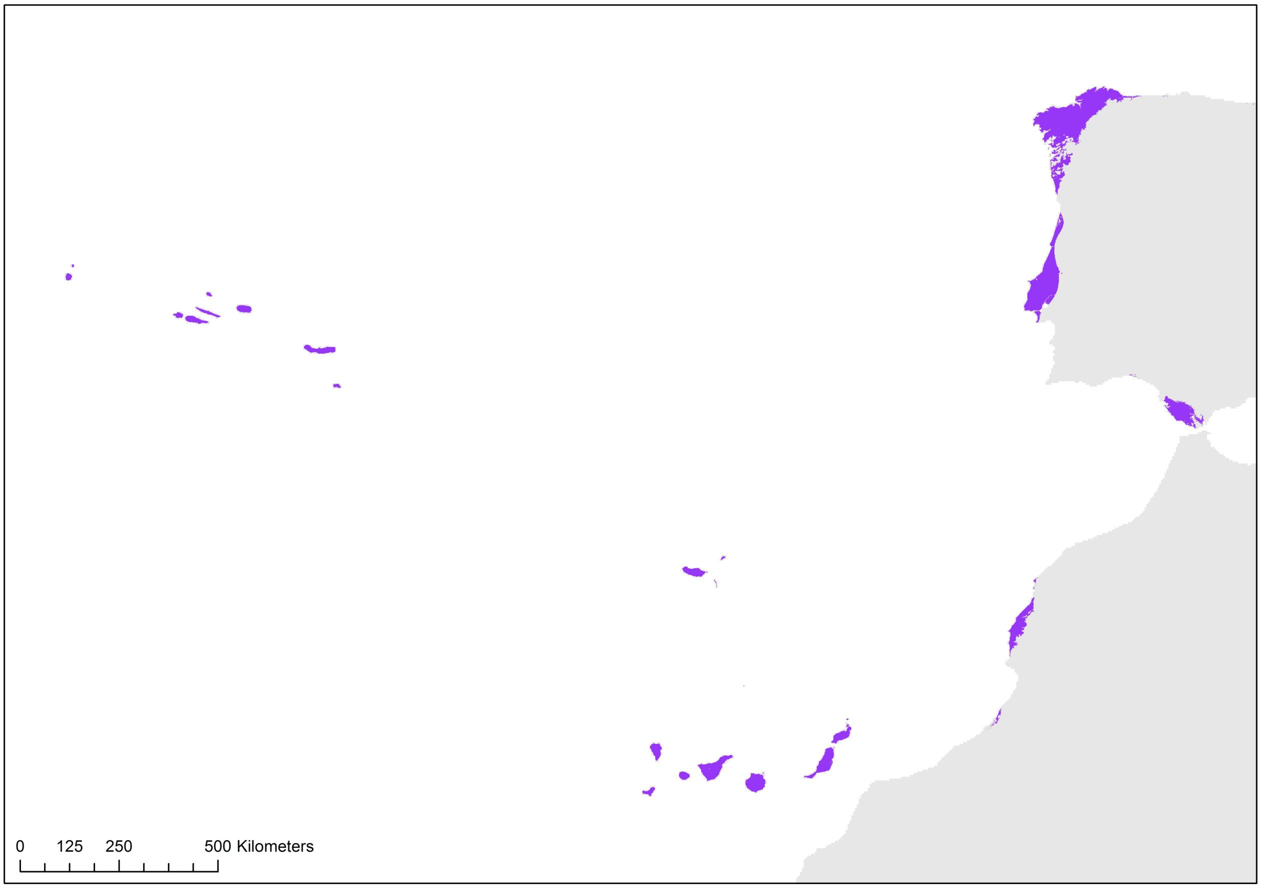

Figure S4. Modelled potential ranges of 35 Macaronesian endemic bryophytes species at present (a) and 2070 under the contrasted climate scenarios defined by the RCP 4.5 (b) and RCP 8.5 (c) concentration pathways. The color scale represents the number of species for which macroclimatic conditions are defined as suitable for a given pixel. The details of the legend are provided in Figure 1. The polygons for the protected areas are reprensetated for Azores, Madeira and the Canaries. Maps were created using ArcGIS software by Esri (Environmental Systems Resource Institute, ArcGIS 10.0; www.esri.com).

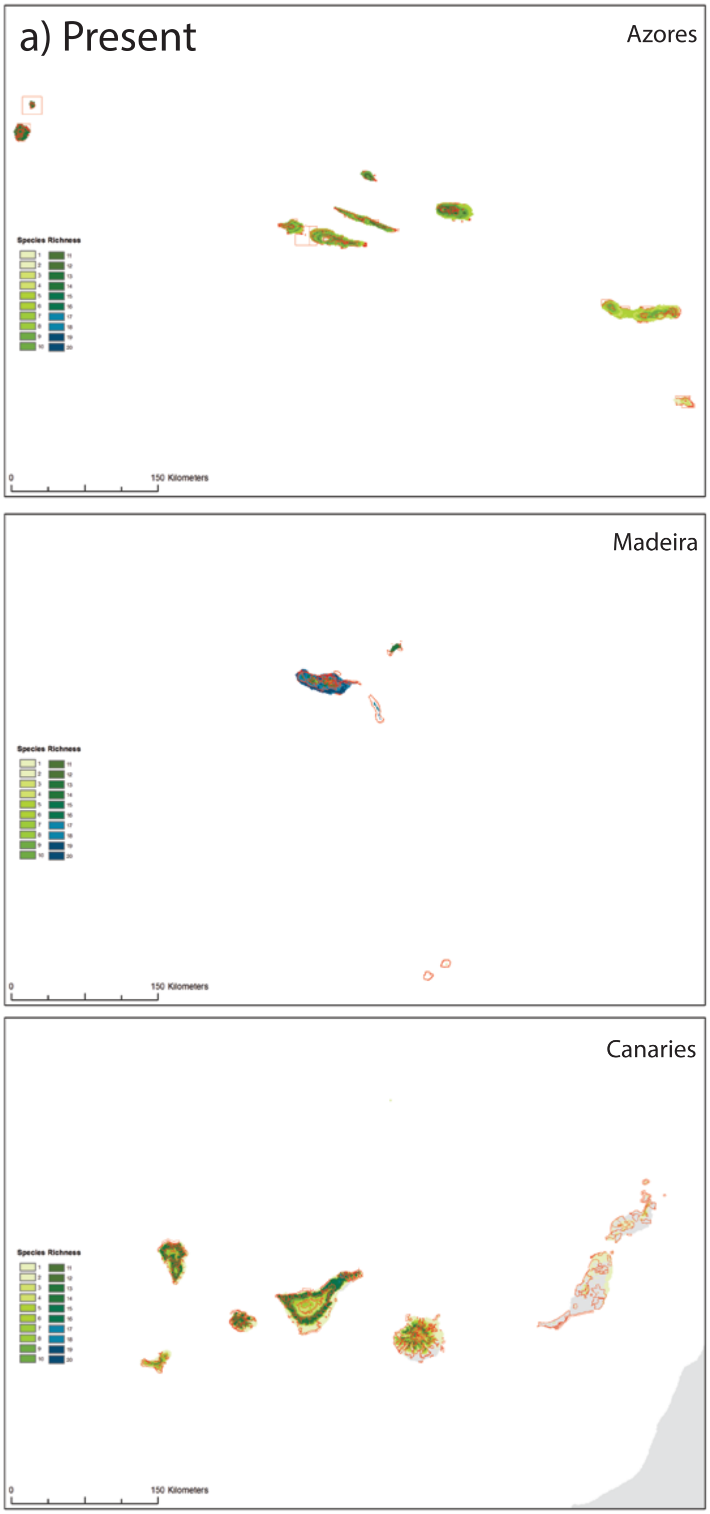

Figure S4 (continued). Maps were created using ArcGIS software by Esri (Environmental Systems Resource Institute, ArcGIS 10.0; www.esri.com).

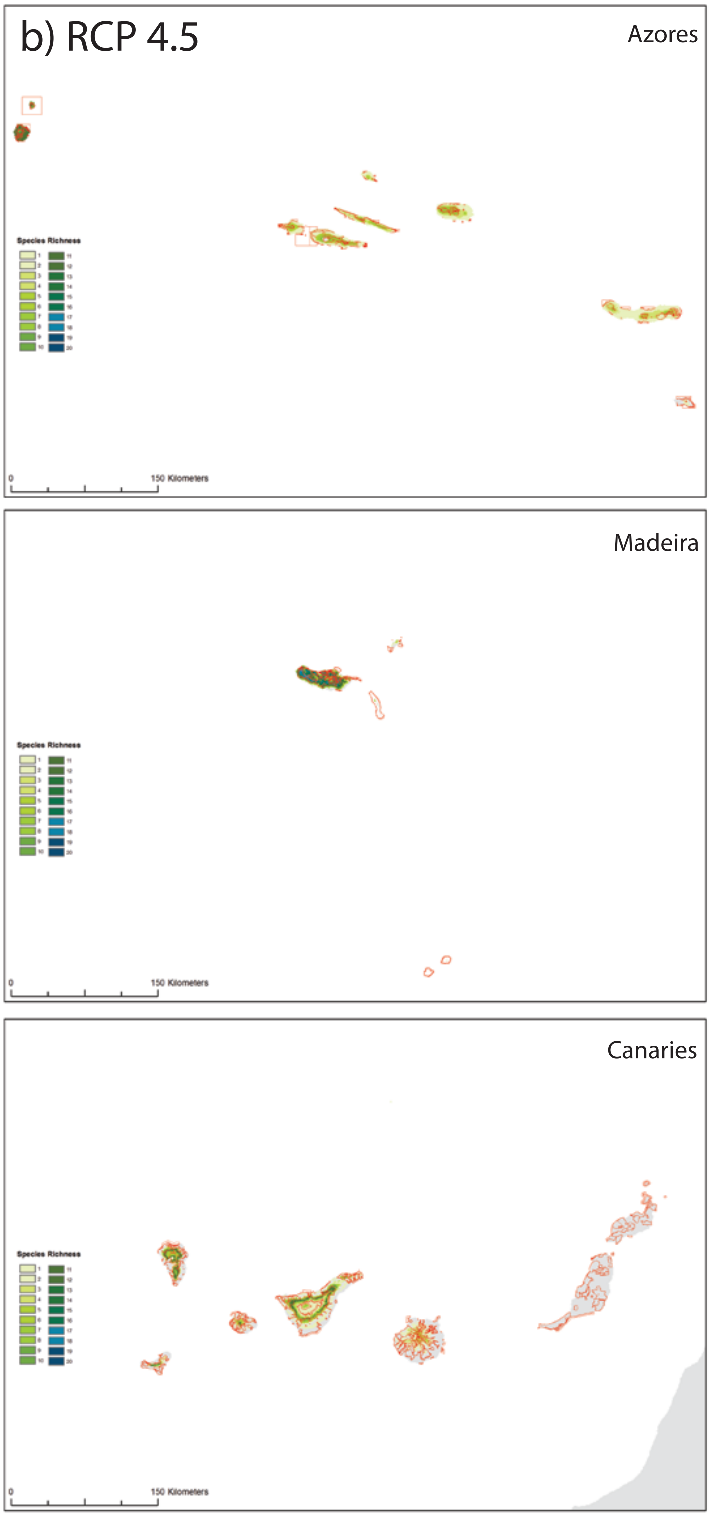

Figure S4 (continued). Maps were created using ArcGIS software by Esri (Environmental Systems Resource Institute, ArcGIS 10.0; www.esri.com).

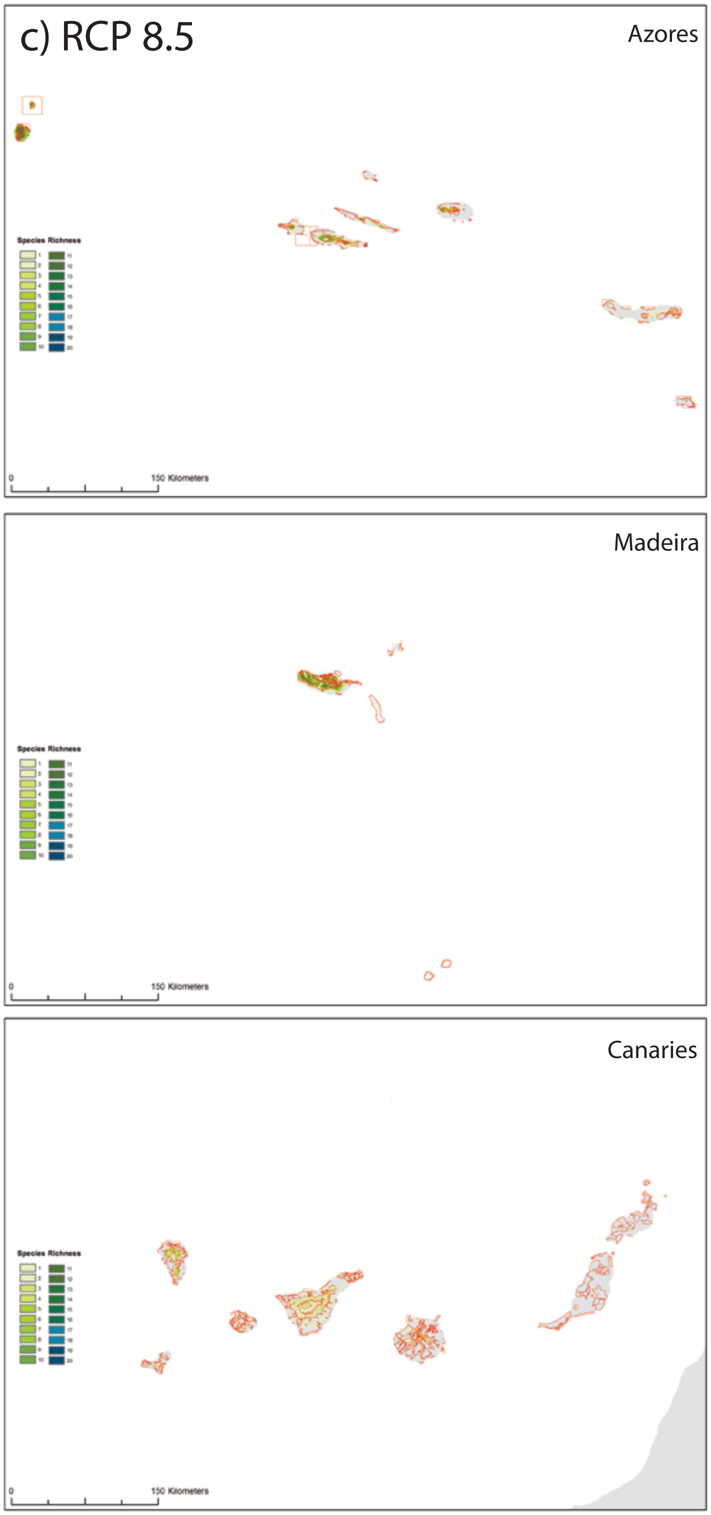

Supplement: Supplementary Information [file srep29156-s1.pdf]
